# ERG inhibition selectively sensitizes ERG-positive prostate cancer to radiation through DNA repair vulnerabilities and enhances response to androgen receptor inhibitors

**DOI:** 10.1186/s13046-026-03854-9

**Published:** 2026-10-23

**Authors:** Ayham Moustafa, Mohamed E. Elsesy, Bente Siebels, Jan Hahn, Su-Jung Oh-Hohenhorst, Carlotta Schudy, Thorsten Rieckmann, Susanne Burdak-Rothkamm, Derya Tilki, Tobias Maurer, Cordula Petersen, Kai Rothkamm, Gunhild von Amsberg, Carsten Bokemeyer, Hartmut Schlüter, Sabrina Köcher, Wael Y. Mansour

**Affiliations:** 1 Department of Radiotherapy and Radiooncology, University Medical Center Hamburg-Eppendorf, Hamburg, Germany; 2 Mildred Scheel Cancer Career Center HaTriCS4, University Medical Center Hamburg-Eppendorf, Hamburg, Germany; 3 Section of Mass Spectrometric Proteomics, University Medical Center Hamburg Eppendorf, Hamburg, Germany; 4 Pharmacology and Experimental Oncology Unit, Cancer Biology Department, National Cancer Institute, Cairo University, Cairo, Egypt; 5 Martini-Klinik Prostate Cancer Center, University Medical Center Hamburg-Eppendorf, Hamburg, Germany; 6 Department of Otorhinolaryngology, University Medical Center Hamburg-Eppendorf, Hamburg, Germany; 7 Department of Molecular & Clinical Cancer Medicine, University of Liverpool, Liverpool, UK; 8 Department of Urology, University Hospital Hamburg-Eppendorf, Hamburg, Germany; 9 Department of Urology, Koc University Hospital, Istanbul, Türkiye; 10 Department of Oncology, II Med. Klinik, Hematology, BMT With Section Pneumology, University Cancer Center Hamburg, University Medical Center Hamburg-Eppendorf, Hamburg, Germany

**Keywords:** Prostate cancer, ERG overexpression, DNA repair, Radiosensitization, ERG inhibition

## Abstract

**Background:**

ERG overexpression, driven by TMPRSS2-ERG gene fusion, occurs in ~ 50% of prostate cancers (PCa) and defines a molecular subtype with distinct therapeutic vulnerabilities. Radiotherapy (RT) is a cornerstone for localized and locally advanced PCa, yet substantial number of patients experience treatment failure. This study investigates whether direct ERG inhibition selectively impairs the resolution of DNA damage signaling and radiosensitizes ERG-positive PCa, providing a precision medicine strategy.

**Methods:**

ERG-positive VCaP cells, ERG-negative DU145/BPH1 cells, ex vivo patient PCa tissue slice cultures (TSCs, *n* = 14 from 10 patients), and patient-derived organoids (PDOs, *n* = 2) were treated with ERG inhibitors (ERGi-USU, VPC-18005) or ERG siRNA ± irradiation (2–6 Gy). DNA double-strand break (DSB) repair was assessed via *γ* H2AX/53BP1 immunofluorescence and pEJ-GFP reporter assay. Clonogenic survival was evaluated in 2D/3D assays. Label-free quantitative LC–MS/MS proteomics identified differentially regulated pathways, validated by immunoblotting. Promoter activity was measured using GLuc-ON™ Promoter Reporter plasmids.

**Results:**

Here, we show that direct ERG inhibition selectively delays the resolution of radiation-induced DSB-associated damage signals and reduces end-joining activity in ERG-positive models, leading to enhanced radiosensitivity. In ERG-positive VCaP cells, ex vivo patient PCa tissue slices, and patient-derived PCa organoids, treatment with ERG inhibitors or ERG-targeted siRNA significantly increased residual *γ*H2AX/53BP1 foci and reduced clonogenic survival following irradiation, whereas ERG-negative tumors remained unaffected. Quantitative LC–MS/MS proteomics, confirmed by immunoblotting and promoter activity assay, revealed that ERG inhibition downregulates key PARP1-mediated end-joining pathway components, including PARP1, LIG3, XRCC1, and the end-resection factor MRE11. Functional assays showed that ERG and PARP inhibition converge on a common mechanism to block alternative end joining, whereas combined ERG and androgen receptor (AR) pathway inhibition further suppressed growth in ERG-positive PCa spheroids and PDOs, probably through metabolic reprogramming.

**Conclusions:**

These data position ERG as a master regulator of DNA repair vulnerabilities in PCa and provide a strong mechanistic rationale for clinical evaluation of ERG-directed radiosensitization and combination therapy strategies in molecularly defined ERG-positive disease.

**Graphical Abstract:**

**Figure:**
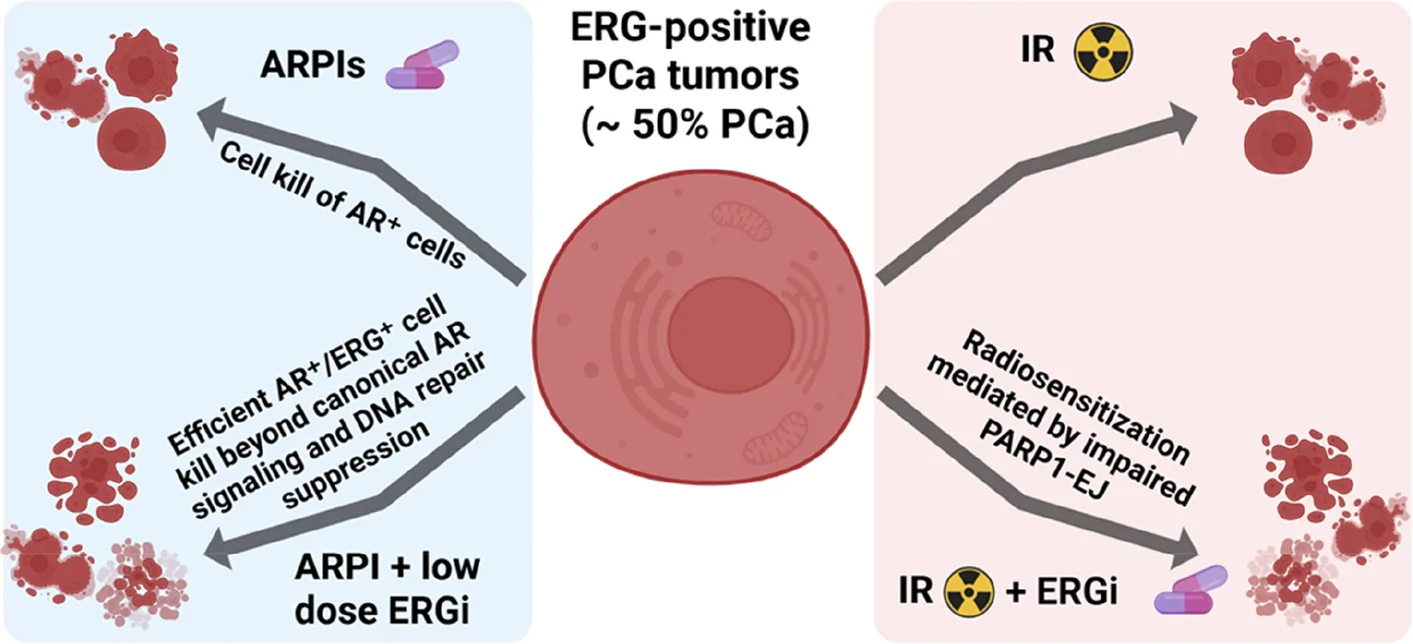

**Supplementary Information:**

The online version contains supplementary material available at https://doi.org/10.1186/s13046-026-03854-9.

## Background

Prostate cancer (PCa) represents the second most common cancer in men worldwide and remains a leading cause of cancer-related mortality [1]. While early-stage localized PCa generally has favorable outcomes, advanced and metastatic disease poses significant therapeutic challenges, particularly due to the development of treatment resistance and disease progression despite multimodal interventions [2]. Radiotherapy (RT) serves as a cornerstone treatment for localized and localized advanced PCa, used as primary therapy, or as adjuvant or early salvage treatment after surgery. In addition, RT plays an important role in the palliative management of PCa, both for effective pain control and for the prevention of metastasis-related complications. The efficacy of RT can be improved when combined with systemic treatment approaches, such as androgen deprivation therapy (ADT) and androgen receptor signaling pathway inhibitors (ARPIs). However, approximately one-third of radiation patients experience treatment failure specifically with definitive RT ± ADT, as evidenced by positive biopsies following therapy [2–4], highlighting an urgent need for strategies to improve long-term RT efficacy. The success of RT decreases with advancing local disease stage and is fundamentally related to the cellular radiosensitivity, which is primarily determined by the capacity to repair ionizing radiation-induced DNA double-strand breaks (DSBs) [5]. DSB repair occurs through two principal mechanisms: non-homologous end joining (NHEJ) and homologous recombination (HR) [5]. However, tumor cells often display dysregulation in classical repair pathways, instead utilizing alternative repair mechanisms such as PARP1-dependent end joining (PARP1-EJ) [6]. Our previous work demonstrated a repair pathway switch to PARP1-EJ in ERG-overexpressing PCa, resulting in selective radiosensitization with olaparib treatment and establishing ERG upregulation as a predictive biomarker for PARP inhibitor plus RT combinations [7].

The discovery of recurrent gene fusions has fundamentally transformed our understanding of PCa's molecular landscape. Genomic fusions between the androgen-regulated transmembrane protease serine 2 gene (TMPRSS2) and E-twenty-six (ETS) transcription factors represent one of the most prevalent alterations in PCa. Among various ETS family members, ERG is the most frequent fusion partner of TMPRSS2, leading to aberrant overexpression of the ERG transcription factor under control of the androgen-driven TMPRSS2 promoter in approximately 50% of PCa cases [8–10]. This makes TMPRSS2:ERG fusion one of the most common genetic aberrations in human cancer. ERG overexpression defines a distinct molecular subtype with unique biological characteristics, yet its role in PCa prognosis remains controversial. While some studies report no or inverse associations between ERG status and clinical outcomes, others demonstrate that ERG overexpression affects disease progression, proliferation, invasion, and tumor aggressiveness [11–16].

In this study, using comprehensive in vitro cell line models, ex vivo tissue slice cultures, and patient-derived organoids, combined with liquid chromatography-tandem mass spectrometry (LC–MS/MS)-based differential quantitative proteomics, we demonstrate that ERG inhibition selectively suppresses DNA DSB repair in ERG-positive PCa cells through downregulation of key PARP1-EJ pathway components, including PARP1, LIG3, XRCC1, and the end-resection protein MRE11. Additionally, we also show that combined inhibition of ERG and AR signaling leads to enhanced growth suppression in ERG-positive models, potentially mediated by metabolic reprogramming. These findings support ERG-guided patient stratification to inform radiosensitization and rational combination therapy strategies.

## Materials and methods

### Cell lines, reagents, and treatment

DU145 and BPH1 cells were cultured in DMEM, HF184 cells in AMnioMax-C100, and VCaP cells in RPMI-1640, all supplemented with L-glutamine, sodium bicarbonate, 10% FBS, and 1% penicillin–streptomycin. Cells were maintained at 37 °C with 5% CO2 and regularly tested for Mycoplasma. Irradiation was performed as described (200 kV, 15 mA, 0.5 mm Cu filter, 0.8 Gy/min) [17]. ERG was inhibited using ERGi-USU (Tocris Bioscience) at concentrations between 0.1 and 0.8 µM, and VPC-18005 (MedChemExpress) at concentrations of 25 and 50 µM). Olaparib, (Selleckchem), 5 µM), was used as a PARP inhibitor. ATM and DNA-PKcs were inhibited with KU-55933 (Tocris Bioscience) at concentrations of 10 µM and NU7026 (Sigma-Aldrich) at concentrations of 10 µM), respectively. Abiraterone and Apalutamide (MedChemExpress (Monmouth Junction, NJ, USA) were used at the indicated concentration to inhibit the androgen.

### Western blot

Western blotting was performed using whole cell lysates as previously described [18]. The following antibodies were used: anti-ERG monoclonal antibody (9FY, mouse; Biocare Medical), anti-PARP1 monoclonal (mouse; BD Biosciences), Poly(ADP-ribose) monoclonal antibody (10H) (mouse; Enzo), Anti-PAR Monoclonal Antibody (rabbit; Trevigen) anti-HSC70 monoclonal (mouse; Santa Cruz), anti-XRCC1 monoclonal (rabbit; Santa Cruz), anti-LIGIII monoclonal (mouse; BD Biosciences), anti-Mre11 monoclonal (12D7, mouse; Invitrogen), PSA/KLK3 (D6B1) Rabbit Monoclonal Antibody (rabbit; cell signaling), Anti-Human Androgen Receptor monoclonal antibody (mouse; BD Pharmingen), Anti-β-Actin monoclonal antibody (mouse; Sigma-Aldrich) and GAPDH antibody (FL-335) (rabbit; Santa Cruz).

### siRNA transfection

VCaP cells were seeded in 6-well plates and transfected with 50 nM of ON-TARGETplus Human ERG (2078) siRNA-SMARTpool (Dharmacon; Cat No. L-003886–00–0005), using TurboFect™ reagent (ThermoFisher Scientific) according to Manufacturer’s protocol. Briefly, transfection complexes were added directly to cells in complete growth medium, followed by 4–6 h incubation at 37 °C/5% CO₂. Medium was then refreshed and cells were incubated for 48–72 h to achieve maximal ERG knockdown prior to irradiation or downstream assays.

### EdU-based cell cycle analysis

EdU-based cell cycle analysis was performed using the EdU-click 488 kit (BCK-EdU488-1, Baseclick GmbH) as previously described [19]. Briefly, cells were treated with or without ERGi (250 nM ERG in 2D, or 150 nM in 3D) for 48 h prior to mock or irradiation (2 Gy in 2D or 3 Gy in 3D). In 2D cultures, cells were pulsed with EdU for 30 min at 37 °C, whereas 3D-grown VCaP spheroids were labelled with EdU for 6 h following irradiation and subsequently dissociated into single cells. Cells were then fixed with 4% paraformaldehyde, permeabilized with 0.5% Triton X-100 in PBS, EdU incorporation was detected using a fluorescent azide-based click chemistry reaction according to the manufacturer’s instructions. DNA was stained with a propidium iodide solution containing RNase. Subsequent analysis of cell cycle distributions was conducted via flow cytometry (FACS CANTO 2, BD Bioscience Systems, Heidelberg, Germany) and Mod-Fit software (Verity Software House).

### Ex vivotissue slice cultures

Tissue slice cultures were prepared from punch biopsies after radical prostatectomy, following our previously published protocol [20]. Ethical approval for these experiments was granted by the Ethics Committee of the Chamber of Physicians of the State of Hamburg (Approval No. PV-3652).

### PDO cultures

Organoid cultures were prepared and maintained according to our previously established methodology [21].

### 3D Cell viability assay

Cell viability in 3D cultures was determined using the CellTiter-Glo® 3D Cell Viability Assay (Promega) according to the manufacturer’s instructions. Briefly, PCa PDOs were dissociated and seeded in a density of approximately 1500 cells per 5 µM dome consisting of a 50% basement membrane extract (BME) in 96-well plates. After BME polymerization for approximately 30 min at 37 °C, each well was overlaid with 100 µL of complete organoid medium. 48 h after seeding, PDOs were treated with ERGi at 150 nM (IC10), either alone or in combination with ARPIs: Abiraterone at 0.1, 0.25, 0.5, and 1 µM, or Apalutamide at 0.1, 0.5, 1, 10, and 50 µM. After 48 h, the ERGi-containing medium was removed and replaced with fresh medium containing the corresponding ARPi concentration only. Organoids were then cultured for an additional 7 days in the continuous presence of the respective ARPi. At the endpoint, an equal volume of CellTiter-Glo® 3D reagent was added to each well, and the plate was shaken for 10 min at 1500 rpm to facilitate lysis and ATP release. After incubation at room temperature for 30 min to stabilize the luminescent signal, luminescence was measured using a Tecan Infinite® F200 PRO microplate reader in luminescence mode with an integration time of 1 s per well. Background signal was subtracted using cell-free control wells, and viability was expressed relative to the corresponding control condition.

### Colony formation assay (CFA)

CFA was used to evaluate cell survival. For 2D monolayer CFAs, exponentially growing cells were treated with DMSO or ERGi for 48 h, irradiated, and then seeded (200 cells/well). After 2–3 weeks, colonies of ≥ 50 cells were fixed in 70% ethanol, stained with 0.1% crystal violet and counted to calculate surviving fractions. For soft agar CFAs, cells were treated with DMSO and ERGi for 48 h, and/or olaparib or ATMi for 1 h before irradiation. VCaP (2500 cells/well) and DU145 (1500 cells/well) were suspended in agarose and growth medium, added onto solid agar layers. To assess the therapeutic efficacy of ERGi in combination with ARPIs, VCaP, DU145, and LNCaP spheroids were treated with increasing concentrations of abiraterone or apalutamide (0.01, 0.1, 0.25, 0.5, and 1 μM), either as monotherapy or in combination with 50 nM ERGi. Colonies were allowed to grow for 3–4 weeks before being stained with MTT, imaged, and quantified using ImageJ.

Tumor organoids survival was assessed with three-dimensional (3D) Matrigel CFAs as previously described [21]. Briefly, single cells were mixed with cold Matrigel (4000 cells/dome), treated as indicated, and incubated for 4 weeks. At the experimental endpoint, organoids were stained with MTT, washed, harvested, and imaged. Survival fraction was determined by ImageJ.

### Immunofluorescence

Immunofluorescence staining and analysis was performed as previously described [21]. Cells were grown on poly-L-lysine-coated coverslips and treated with DMSO, ERGi (48 h), olaparib, or ATMi (1 h) prior to irradiation. For ex vivo assay, TSCs were fixed 24 h after irradiation with 4% PFA, washed in 25% sucrose, embedded in TissueTek, and sectioned into 5 μm cryoslices. Fixed cells or TSCs were permeabilized and incubated with primary these antibodies: γH2AX (Anti-phospho-S139, monoclonal, mouse, Cell Signaling; 1:500), 53BP1 (Anti-53BP1, polyclonal, rabbit, Novus Biologicals; 1:500), ERG (anti-ERG monoclonal antibody, rabbit, abcam); 1:1000), followed by secondary antibodies (Alexa Fluor 594 anti-mouse, Alexa Fluor 488 anti-rabbit; 1:500). DNA was counterstained with DAPI (10 ng/mL). Immunofluorescence staining of PDOs following ERGi treatment was performed as previously described [22]. Briefly, PCa organoids were treated with ERGi for 48 h, and irradiated with 3 Gy or left untreated. Organoids were harvested 1 or 24 h later, fixed in 4% PFA, cryoprotected in sucrose, embedded in OCT, cryosectioned at 5 µm, and stained for γH2AX and 53BP1. Images were acquired using a Zeiss AxioObserver.Z1 microscope, with Z-stack imaging via Zeiss Apotome and ZEN 3.5 software. DNA double-strand break foci were quantified by counting foci in at least 100 cells using ImageJ for both cell lines and tumor samples.

### Scoring micronucleated cells

Micronucleated cells were counted manually from DAPI stained cells using fluorescence microscopy. Cells were fixed with 4% paraformaldehyde, stained with DAPI, and mounted in antifade medium. Micronuclei were identified according to standard morphological criteria as distinct, round or oval DAPI-positive structures separated from the main nucleus, with a diameter smaller than one-third of the main nuclear diameter. Only interphase cells with clearly distinguishable nuclei were scored, whereas overlapping cells, mitotic cells, and apoptotic bodies were excluded from analysis. For each experimental condition, at least 500 cells were evaluated per replicate in a blinded manner, and the frequency of micronucleated cells was calculated as the percentage of cells containing one or more micronuclei.

### Monitoring of lethal chromosomal aberrations

Lethal chromosomal aberrations were analyzed in metaphase spreads after treatment as previously described [23]. Briefly, treated cells were exposed incubated with Colcemid (Demecolcine, Sigma-Aldrich) to arrest cells in metaphase, harvested, and subjected to hypotonic swelling followed by fixation in methanol/acetic acid. Cell suspensions were dropped onto clean microscope slides, air-dried, and stained with Giemsa or an equivalent chromosomal stain. Metaphase spreads were examined by light microscopy, and only well-spread metaphases with clearly separated chromosomes were scored. Chromosomal aberrations were evaluated according to standard cytogenetic criteria. Dicentric chromosomes, acentric fragments, and chromosome breaks were recorded as lethal aberrations. A total of 246 metaphases were analyzed in a blinded manner.

### Neutral comet assay

Detection of DNA DSBs was performed using a comet assay kit (Trevigen, cat. no. 4250–050-K) as previously described [24], according to the manufacturer’s instructions. Briefly, following treatment, cells were harvested and mixed with low-melting-point agarose at a 1:100 ratio, then transferred onto Comet Slides. After lysis, electrophoresis was carried out at 1 V/cm for 35 min. Slides were stained with SYBR Gold (Invitrogen, cat. no. S11494) for 30 min and imaged using a Zeiss AxioObserver.Z1 microscope, with an FITC filter. DNA tail length and intensity were quantified as tail moment using OpenComet plugin within ImageJ software.

### GLuc-ON™ promoter reporter assay

Commercially available GLuc-ON™ reporter constructs (GeneCopoeia) containing human promoter for *PARP1* (HPRM43771-PF02), *LIG3* (HPRM44239-PF02), *XRCC1* (HPRM70945-PF02), or *GAPDH* (GAPDH-PF02), cloned upstream of eGFP in the pEZX-PF02 vector were used for promoter assays. Cells were transfected with the individual plasmids using TurboFect (Thermo Fisher Scientific) according to the manufacturer’s instructions. Forty-eight hours post-transfection, cells were harvested, fixed, and the percentage of GFP-positive cells was quantified by flow cytometry (FACSCanto II, BD Biosciences, Heidelberg, Germany) using ModFit LT software (Verity Software House).

### Sample preparation for proteomic analysis

For LC–MS/MS-based proteomics, cells at 70–90% confluence in exponential growth, were harvested for quantitative proteomic analysis. After five gentle washes with ice-cold PBS, cell pellets were dissolved in 300 µl triethylammonium bicarbonate buffer (TEAB) with 1% sodium deoxycholate (SDC) for proteins extraction**.** Snap frozen patient tissues were sampled and homogenized using a nanosecond infrared laser (NIRL) (Fig. S7A). The ablation setup and laser parameters were used as in previous work [25]. Briefly, the snap frozen patient tissues were placed on cooling stage (−10 °C) in closed ablation chamber. Based on the laser ablation, the generated aerosol in volume of 0.25 µl of each replicate was collected on a glass slide facing the ablation side and finally solubilized in 1% SDC/TEAB buffer and used for subsequent sample preparation. The proteins from cell lines and tissues were heated at 95 °C for 5 min to denature proteins, followed by sonication to shear nucleic acids. To reduce disulfide-bonded cysteine residues, 10 mM dithiothreitol (DTT) was added to the samples, followed by incubation for 30 min at 60 °C. Subsequently, 20 mM iodoacetamide (IAA) was added for alkylation of reduced cysteines, and the samples were incubated for 30 min at 37 °C in the dark. Proteins were digested with trypsin (1:100 ratio) at 37 °C for 16 h. Trypsin activity was quenched and SDC precipitated with formic acid (final 1% v/v). After 5-min centrifugation at 16,000 g, the peptide-containing supernatants were transferred to clean tubes and dried in a SpeedVac vacuum concentrator.

### Mass spectrometric analysis

Bottom-up differential quantitative proteomics was conducted using nano-UPLC (Dionex UltiMate 3000) coupled to a Quadrupole Orbitrap hybrid mass spectrometer (QExactive, Thermo Fisher Scientific, Waltham, MA, USA). Tryptic peptides were resuspended in 0.1% formic acid (1 µg/µl), and 1 µl was injected for analysis. All subsequent measurement setups were performed as described previously [7, 25]. Briefly, peptides were desalted on a C18 trapping column and separated on a C18 reversed-phase column. Chromatographic separation of peptides was performed using a two-buffer system (buffer A: 0.1% FA in H2O, buffer B: 0.1% FA in ACN), using a gradient length of 60 min. Eluted peptides were ionized by nano-ESI (1800 V) and analyzed by the QExactive in data-dependent acquisition mode, covering 400–1200 m/z with 70,000 resolution at m/z 200. MS/MS was performed with 25 normalized collision energy and 17,500 resolutions, analyzing the top 15 most intense precursor ions per scan, with an AGC target of 1 × 10^5^ ions and maximum injection time of 50 ms, starting from 100 m/z. Proteomics of VCaP cells treated with ERGi plus ARPIs samples (illustrated in Fig. 7 & Fig. S10 C&D) was conducted as described previously [22] and processed in data-independent acquisition mode DIA.

### LC–MS/MS data processing

DDA and DIA MS raw files of all samples were searched against the reviewed Homo sapiens UniProt database (Dec. 2022, 20,330 entries) using the Sequest algorithm in Proteome Discoverer 2.4 (Thermo Fisher Scientific). Label-free quantification with match between runs was applied. Carbamidomethylation of cysteines was set as a fixed modification, while protein N-terminal acetylation, methionine oxidation, and N-terminal glutamine cyclization were variable modifications. Fragment mass tolerance was 10 ppm, peptides of at least 6 amino acids and up to 6000 Da were accepted, allowing up to two missed trypsin cleavages. Fragment spectra error tolerance was 20 ppm. Peptides were filtered for high confidence, using a false discovery rate (FDR) < 1% based on a decoy database. Peptide precursor areas were summed to protein areas, with only unmodified peptides used for quantification.

### Statistical analysis and visualization

Perseus software (Max Plank Institute for Biochemistry, version 1.5.8.5) was used in statistical analysis [26]. Protein abundances were imported into the software, log2 transformed, and median-normalized by column to overcome the injection-related discrepancies in total protein amounts. Two sample t-tests and ANOVA were performed within the software using a FDR-adjusted *p* < 0.05. KEGG pathway enrichment analysis was performed using clusterProfiler (v4.10.1) [27], with pathway annotations based on KEGG [28]. In addition, Hallmark pathway enrichment was conducted using the MSigDB Hallmark gene set collection [29], which comprises 50 curated Hallmark gene sets that summarize and reduce redundancy across the broader MSigDB. Relative protein abundances among all replicates were conducted using two-sided Wilcoxon rank-sum tests implemented in R (Wilcox. Test), and p-values are reported in the corresponding figures. The R software environment (version 4.3.2) was used in data visualization including, heat maps (ComplexHeatmap), volcano plots and bubble charts of the enriched terms. Bar plots and survival fractions graphs were visualized in GraphPad Prism 8.0.2. The graphical abstract was created in BioRender. Unless otherwise specified, the intensities of the western blot bands were detected, measured and quantified using Licor Odyssey CLx imaging system. Western blot results were normalized to HSC70 or beta actin or GAPDH and are presented as means ± SEM from three independent experiments. All experiments were conducted at least three times using distinct biological replicate samples. Statistical significance was defined as p-value of *P* < 0.05 for *; *p* < 0.001 for **; and *p* < 0.0001 for ***; ns: not significant.

## Results

### ERG inhibition impairs DNA DSB repair in ERG-positive PCa cells

Recently, we provided substantial evidence supporting the rationale for targeting ERG-overexpressing PCa cells with PARPi as radiosensitisers [7]. These findings strongly suggest that direct targeting of ERG may be an effective and highly selective strategy to enhance radiosensitivity in ERG-positive PCa. To investigate this, we employed the previously characterized ERG inhibitor ERGi-USU [30] to suppress ERG expression in ERG-positive VCaP cells, using ERG-negative DU145 and BPH1 cells as controls (Fig. S1A). To determine optimal dosing, we tested ERGi concentrations ranging from 0.1 to 0.8 µM for up to eight days, refreshing the media every 48 h. The IC₅₀ values were 0.217 µM for VCaP, 0.433 µM for DU145, and 0.363 µM for BPH1 (Fig. S1B). Consistent with prior data, treatment with 0.25 µM ERGi for 48 h markedly reduced ERG protein levels by nearly 50% in ERG-positive VCaP cells (Fig. S1C). Notably, ERG expression began to recover by 72 h and was nearly fully restored by 120 h post-treatment (Fig. S1D), indicating that 0.25 µM for 48 h is effective for transient ERG suppression, but sustained inhibition requires repeated dosing.

**Fig. 1:**
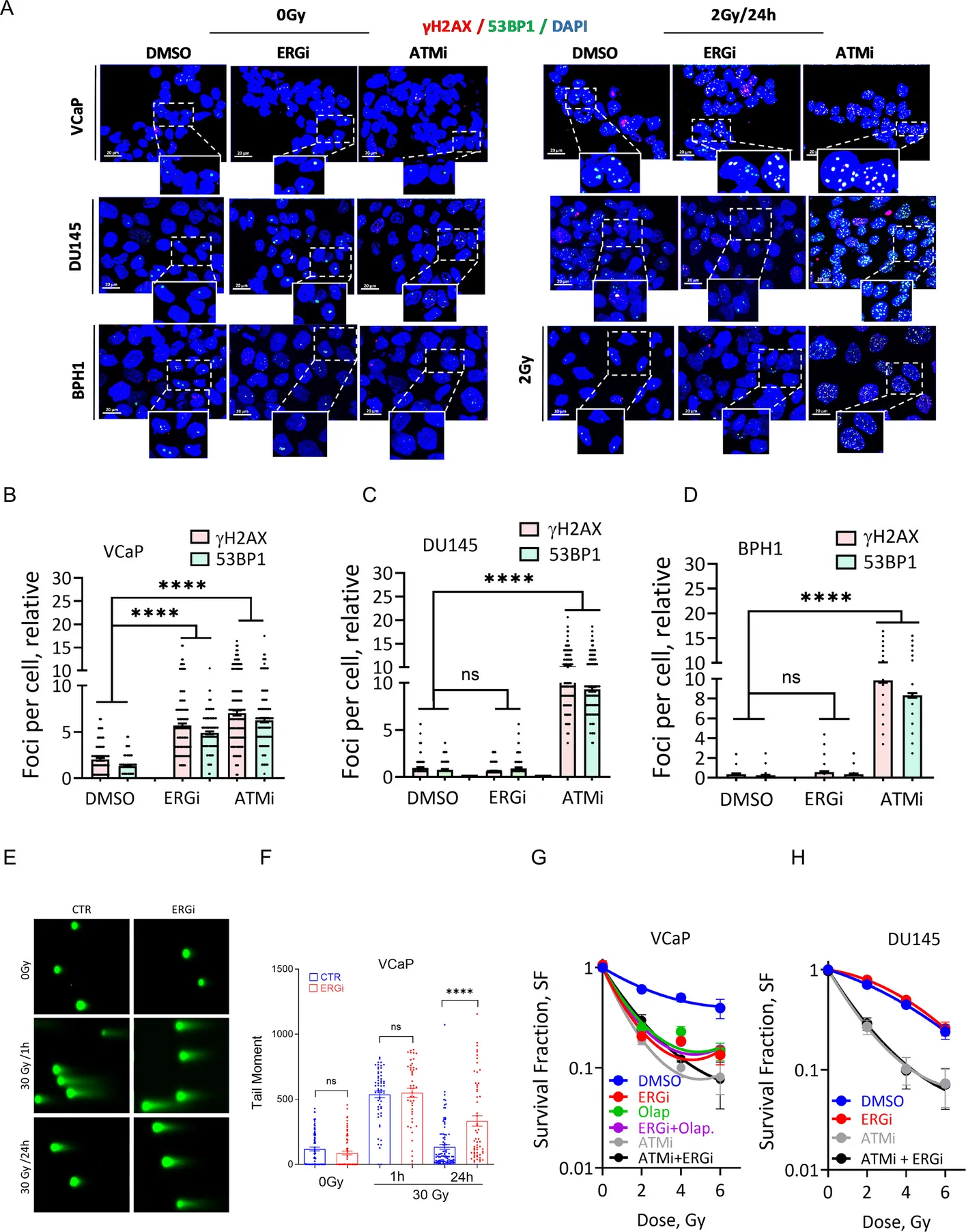
ERGi selectively radiosensitizes ERG-positive PCa cells by impairing DSB damage signaling. **A** Representative IF images of gH2AX (red) and 53BP1 (green) under the indicated treatment conditions in VCaP, DU145, and BPH1 cells. Scale: 20 µm. **B-D** Quantification of gH2AX/53BP1-foci in **B** VCaP, **C** DU145 and **D** BPH1 cells. For the indicated treatments, relative foci numbers were calculated using as a reference the foci observed following each treatment alone without irradiation. **E** Representative images of the comet assay in VCaP cells treated with vehicle control (CTR) or ERGi, followed by irradiation (30 Gy). Cells were collected at 1 and 24 h post irradiation. **F** Quantification of comet assay in E. **G** & **H** Survival fractions measured by 3D-colony forming assay of irradiated (0, 2, 4, 6 Gy) (G) VCaP and (H) DU145 pre-treated with ERGi and ATMi, either individually or combined. Shown are mean ± SEM of at least six independent experiments. Statistical significance was determined using an unpaired Mann–Whitney t-test, ****p < 0.0001. ns: not significant

We next assessed the effect of ERG inhibition on the resolution of IR-induced DNA damages. Therefore, VCaP, DU145, and BPH1 cells were pre-treated with 0.25 µM ERGi and analyzed for *γ*H2AX and 53BP1 foci, surrogate markers of DSBs, at 1 h and 24 h after 2 Gy irradiation (Fig. 1A). While no significant differences were observed at 1 h (Fig. S2A), ERG-inhibited VCaP cells exhibited a significant increase in residual *γ*H2AX and 53BP1 foci at 24 h compared to DMSO-treated controls (*p* < 0.0001, Fig. 1B). Kinetics analysis revealed that ERG inhibition did not affect the initial induction of γH2AX and 53BP1 foci after irradiation, but significantly delayed their resolution, resulting in persistent foci at 12–48 h and indicating delayed resolution of radiation-induced DSBs-signaling (Fig. S2B). In contrast, ERG-negative DU145 and BPH1 cells showed no significant changes in foci numbers following ERG inhibition (*p* = 0.403 and *p* = 0.111, respectively) (Fig. 1C & D). As expected, ATM inhibition, used here as a positive control for impaired DDR and DSB repair, led to increased residual foci in all three cell lines (Fig. 1B-D). Comet assay confirmed the foci data, showing that ERG inhibition in VCaP cells did not markedly increase the initial radiation-induced DNA damage, but instead delays its resolution over time (Fig. 1E&F). ERGi treatment for 48 h did not induce significant cell-cycle arrest in either 2D or 3D VCaP cultures (Fig. S2C&D). Because VCaP cells harbor mutant TP53 that precludes canonical G1/S arrest following DNA damage, both control and ERGi-treated models exhibited a transient accumulation in S/G2 phase at 24 h in 2D cultures and recapitulated at 48 h in 3D spheroids. Notably, ERGi-treated cells and spheroids mounted a substantially more pronounced and prolonged S/G2 enrichment than controls. This was evident at 24 h (54.7% vs 40.10%) in 2D cultures and recapitulated at 48 h in 3D spheroids (45.6% vs 33.0%) (Fig. S2C&D), consistent with impaired DSBs signaling and delayed checkpoint recovery. Importantly, this delay was transient, with cell-cycle distributions gradually returning toward baseline by 72 h post-irradiation (Fig. S2C&D). Furthermore, ERGi treatment significantly increased radiation-induced lethal chromosomal aberrations (Fig. S2E) and micronucleated cells (Fig. S2F) compared with irradiation alone, whereas ERGi alone did not affect either endpoint. These data suggest that ERG inhibition sensitizes cells to ionizing radiation by exacerbating chromosomal damage and eventually inducing mitotic catastrophe, potentially through compromised DNA damage repair.

To further validate the role of ERG in IR-induced DNA damage siganling, we used another well-characterized ERG inhibitor (VPC-18005) that blocks ERG-DNA binding [31]. Treatment with 50 µM VPC-18005 for 48 h significantly increased *γ*H2AX and 53BP1 foci in VCaP cells at 24 h post-IR (*p* < 0.0001, Fig.S3A&B), despite ERG protein levels remaining unchanged (Fig. S3C). Furthermore, no additive effect was observed when VCaP cells were treated with both VPC-18005 and ERGi-USU (Fig. S3B). Collectively, these data suggest that ERG plays a critical and potentially direct role in regulating DNA damage signaling, and its inhibition selectively impairs this process in ERG-overexpressing PCa cells.

### siRNA-mediated ERG-knockdown confirms ERG’s critical role in DSB repair

To exclude potential off-target effects of pharmacological ERG inhibition by the indicated ERGi, VCaP cells were transfected with a SMARTpool siRNA targeting ERG and analysed at 24, 48, and 72 h post-transfection to determine the optimum silencing time window. As illustrated in Fig. S3D&E, maximal ERG knockdown in VCaP cells was achieved between 48 and 72 h post-transfection. To assess the effect of ERG-knockdown on DSB repair, VCaP cells were treated with siERG for 48 h before being irradiated with 2 Gy, and *γ*H2AX/53BP1 foci were monitored at 1 h and 24 h post-irradiation (Fig. S3F). ERG knockdown led to a significant increase in residual *γ*H2AX and 53BP1 foci (Fig. S3G), mirroring the effects observed following pharmacological ERG inhibition. Importantly, co-treatment with ERGi did not further increase foci numbers compared to siERG alone (Fig. S3G). Together, these data confirm that ERG plays a functional role in regulating DNA DSB repair, and that its targeting selectively disturbs repair in ERG-overexpressing PCa cells. Furthermore, the lack of additive effects between siERG and ERGi further supports an on-target mechanism, with selective impairment restricted to ERG-overexpressing PCa cells.

## ERG inhibition selectively radiosensitizes ERG-positive PCa cells

ERGi-mediated disruption of DNA DSB repair in ERG-overexpressing cells strongly suggests a selective radiosensitization effect. To directly analyze this, VCaP and DU145 cells were treated with ERGi for 48 h before being irradiated with varying X-ray doses (0, 2, 4, 6 Gy) and a 3D soft agar CFA [32] was utilized to assess survival outcomes (Fig. S5A&B). In ERG-positive VCaP cells, ERGi combined with IR significantly inhibited growth compared to the DMSO plus IR control (Fig. 1G). Conversely, ERGi plus IR treatment did not alter colony formation in DU145 cells compared to the corresponding negative control (Fig. 1H). Since benign BPH1 cells failed to form colonies in the 3D culture system, as previously described [33], the effect of ERGi on cellular clonogenic survival post-irradiation was analyzed in a 2D monolayer assay (Fig. S5C). Results revealed that BPH1 exhibited no decrease in cell survival following ERGi + IR treatment (Fig. S5D). Notably, ERGi did not increase the radiosensitivity of the normal human fibroblasts HF186 cells (Fig. S5E), further demonstrating that this combination selectively sensitizes ERG-positive tumor cells while sparing ERG-negative normal and tumor cells. The inhibition of ATM, as a positive control, enhanced the cytotoxic effects of IR across all cell lines (Fig. 1G & H and Fig. S5D) and notably, simultaneous inhibition of ATM and ERG did not show an additive effect compared to individual inhibition. Taken together, these findings suggest that ERG inhibition selectively enhances radiosensitivity in ERG-positive PCa cells, providing potential therapeutic insight for targeted radiosensitization.

### ERG and PARP inhibition converge on a common PARP1-dependent repair pathway to radiosensitize ERG-positive PCa cells

**Fig. 2:**
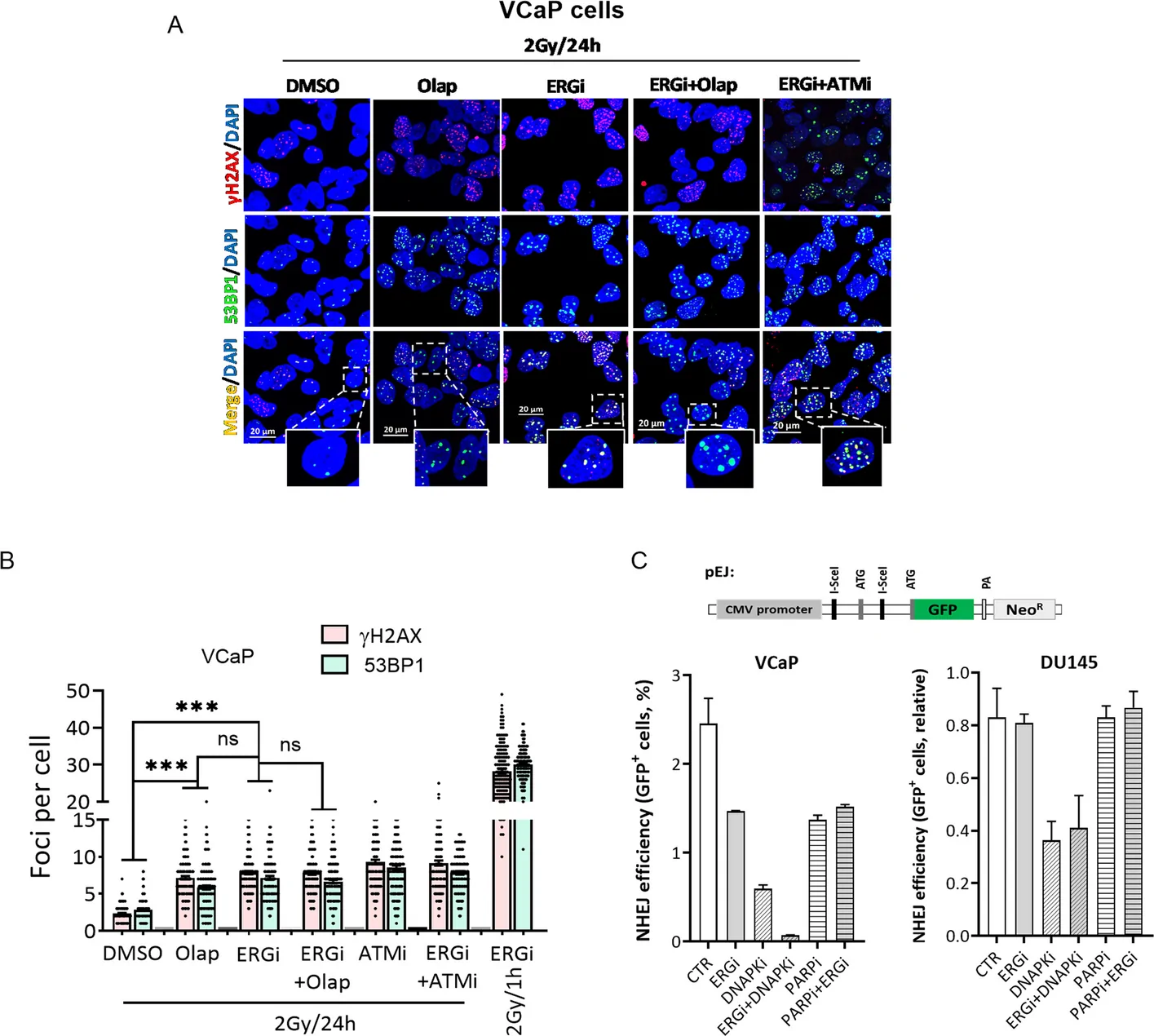
ERG and PARP inhibition act through a shared pathway to radiosensitize ERG-positive PCa cells by impairing PARP1-EJ. **A** Representative immunofluorescence images showing gH2AX (red), 53BP1 (green) in VCaP cells following the indicated treatments. Scale: 20 µm. **B** Quantification of the data shown in B. **C** Upper panel: Schematic representation of the NHEJ reporter construct pEJ, as previously described in [18, 34]. Lower panel: Bar plots quantifying GFP-positive cells resulting from efficient NHEJ of I-SceI-induced DSBs. GFP signals were measured 48 h post-treatment ± ERGi, ± DNA-PKcsi in transfected VCaP and DU145 cells. Transfection efficiency was normalized by co-transfection with the pEGFPN1 plasmid. Data are presented as mean ± SD of two independent experiments. ***p < 0.001. ns: not significant

Previously, we reported that PARP inhibition selectively radiosensitizes ERG-positive PCa cells by impairing DSB repair [7]. Given that ERG inhibition also displays selective effects in ERG-positive cells, we sought to determine whether PARP and ERG function within the same DNA repair pathway to regulate radiosensitivity and DSB repair. To address this, we investigated the effects of ERGi and/or PARPi olaparib on survival and DSB repair in ERG-positive VCaP cells. As illustrated in Fig. 2A&B and S5A, and consistent with our previous findings [7], treatment with either ERGi or olaparib significantly and selectively enhanced radiosensitivity in VCaP cells. Consistently, ERGi or olaparib treatment increased the number of residual *γ*H2AX and 53BP1 foci and reduced the survival fractions at 24 h post-2 Gy in VCaP cells (Fig. 2A &B and S5A). Notably, the combination of ERGi and olaparib did not further reduce cell survival or increase the number of residual DSB foci compared to either treatment alone (Fig. 2A&B and S5A), suggesting that both inhibitors act through a common pathway. Neither ERGi nor olaparib, alone or combined, increased the number of residual *γ*H2AX and 53BP1 foci at 24 h post-2 Gy in ERG-negative DU145 and BPH1 cells (Fig. S4A-C).

We previously demonstrated that DSB repair in ERG-positive PCa cells is switched to the PARP1-EJ pathway, rendering these cells particularly susceptible to radiosensitization by PARP inhibition [7]. This raises the hypothesis that ERGi may exert its selective effect towards the ERG-positive cells by targeting the PARP1-EJ pathway. Supporting this model, combined ERGi and olaparib treatment did not further increase residual *γ* H2AX levels beyond those observed with either treatment alone in VCaP cells. In support of this model, irradiation induced robust PAR signals in ERG-overexpressing VCaP cells, indicating increased PARP activity, and both ERGi and olaparib profoundly attenuated this signal (Fig. S4D), consistent with convergence on a shared PARP1-dependent repair pathway. Notably, olaparib did not alter ERG expression or AR signaling, indicating that the observed effects were not due to changes in androgen receptor pathway activity. To directly test whether ERGi suppresses PARP1-EJ activity, we employed our previously described GFP-based pEJ reporter assay (Fig. 2C, [34]). VCaP and ERG-negative DU145 cells were treated with ERGi, followed by transfection with an I-SceI-linearized pEJ plasmid. End joining (EJ) efficiency was quantified by FACS as the percentage of GFP-positive cells. In VCaP cells, treatment with either ERGi or PARPi reduced GFP signal by ~ 50% with no additional reduction observed upon combining both drugs (Fig. 2C), further supporting a shared, common mechanism of action. On the other hand, inhibition of DNA-PKcs (DNAPKi), a core factor in classical NHEJ, also decreased repair efficiency by ~ 50%, but the combination of ERGi and DNAPKi almost completely abolished EJ activity. This indicates that ERG and DNA-PKcs act in distinct repair pathways. In ERG‑negative DU145 cells, DNAPKi reduced EJ efficiency by ~ 50%, while PARP or ERG inhibition alone had no effect on EJ, and combined DNAPKi plus ERGi did not further decrease EJ activity (Fig. 2C). Together, these findings indicate that ERG inhibition phenocopies PARP inhibition in ERG-positive cells, and that both interventions coverage upon a common DSB repair (*i.e*. PARP1-EJ pathway) and enhance radiosensitivity. This supports the concept of targeting ERG activity as a promising therapeutic strategy for selective radiosensitization in ERG-positive PCa cells.

### Ex vivo validation of the selective ERGi-mediated impaired DSB repair in ERG-positive PCa patient tissues

**Fig. 3:**
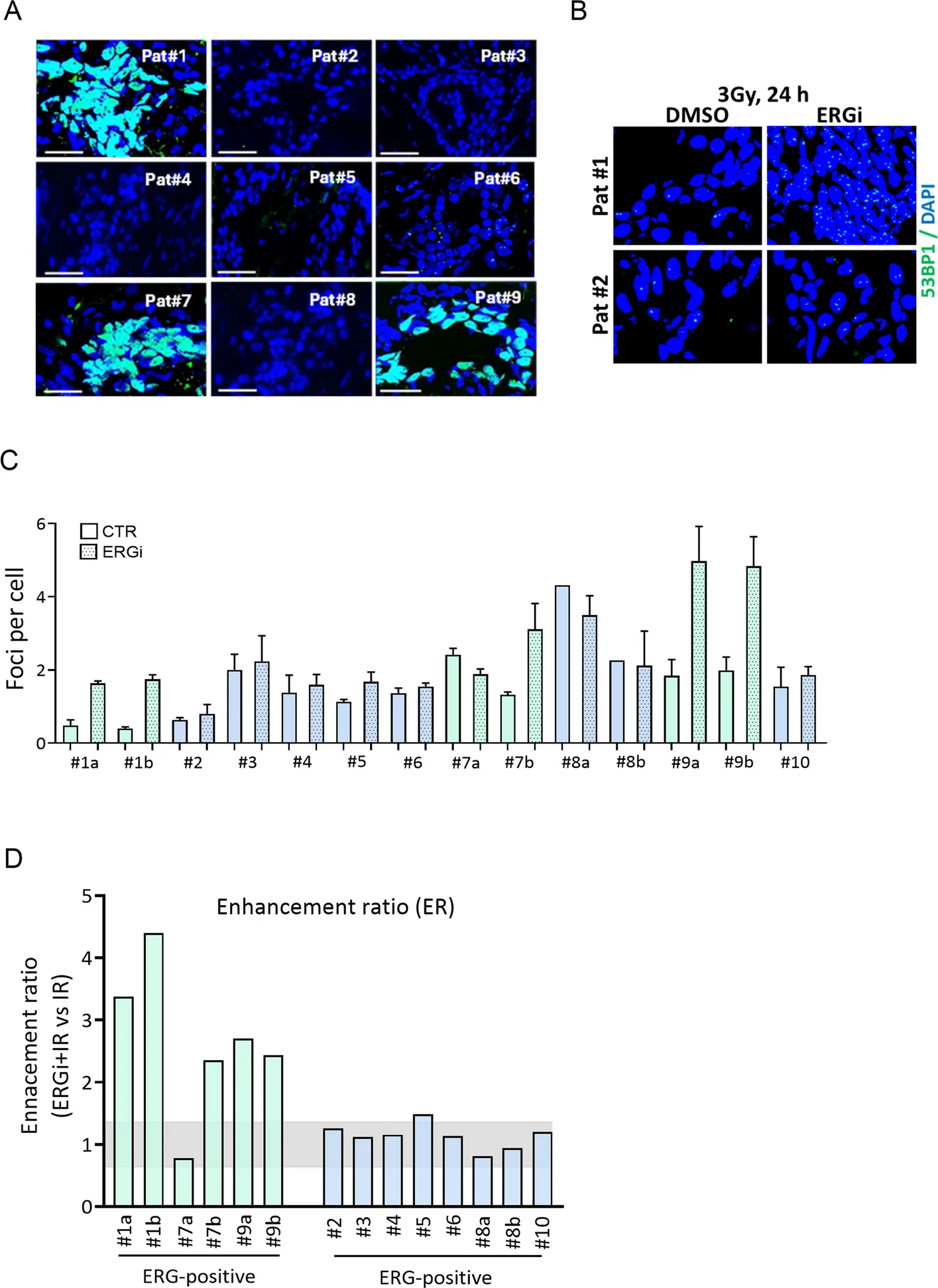
ERG inhibition significantly suppresses the resolution of IR-induced DSBs in ERG-positive PCa patient tissues. **A** Representative IF images showing ERG (green) and DAPI (blue) staining in tissue sections from PCa patients with varying ERG-status. **B** Representative IF images of 53BP1 foci (green) and DAPI (blue) at 24 h post-2 Gy in TSCs from Pat #1 (ERG-positive) and Pat #2 (ERG-negative) PCa patients. **C** Quantification of 53BP1 foci in TSCs from the indicated PCa patients at 24 h post-3 Gy before (DMSO) and after ERG inhibition (ERGi). **D** Enhancement ratio analysis as indicated by fold change of residual 53BP1 foci in TSC treated with ERGi + 3 Gy vs 3 Gy alone, grouped by ERG status

To further validate our findings, we employed our previously established functional ex vivo assay [20] as a preclinical, patient-derived model to assess the effect of ERG inhibition on DSB repair in PCa. For this purpose, 14 punch biopsies were collected from 10 PCa patients, who underwent radical prostatectomy at the Martini-Klinik, and maintained as tissue slice cultures (TSCs) under optimized oxygenated conditions that prevent hypoxia, as previously described [20]. ERG staining revealed that three patients had ERG-positive tumors (Fig. 3A). All TSCs were treated with 0.25 µM ERGi for 48 h prior to irradiation with 3 Gy, and residual 53BP1 foci, as a DSB surrogate marker, were monitored 24 h after IR (Fig. 3B). Variability in the number of 53BP1 foci was observed between individual patients (Fig. 3C). Importantly, ERG inhibition caused a significant increase in residual 53BP1 foci exclusively in TSCs from patients with ERG-positive PCa (Fig. 3C). Consistent with the 53BP1 findings, γH2AX foci mirrored these results, confirming the expected strong concordance between both markers (Fig. S5F).

The ERGi enhancement ratio, calculated as fold change in residual 53BP1 foci with ERGi + IR vs IR alone, was used as a surrogate measure of ERGi-mediated radiosensitization effect. One ERG-positive patient sample (# 7a/b) was ERG-positive in both biopsies, but showed discrepant ERGi + IR responses, with one biopsy classified as a responder and the other as a non-responder. This intra-patient heterogeneity may arise from temporal tumor evolution between biopsies, including clonal selection of ERG-independent subpopulations or acquired changes in downstream signaling pathways or epigenetic modulation of ERG target genes. Except for this case, the TSCs (n = 5/6) from the three ERG-positive patient samples displayed increased ERGi enhancement ratios (Fig. 3D) and were therefore classified as responders. These findings provide ex vivo evidence that ERGi selectively impairs DSB repair in ERG-positive tumors, supporting its potential as a targeted radiosensization strategy.

### ERG inhibition alters the proteome and suppresses DNA repair pathways in ERG-positive PCa cells

**Fig. 4:**
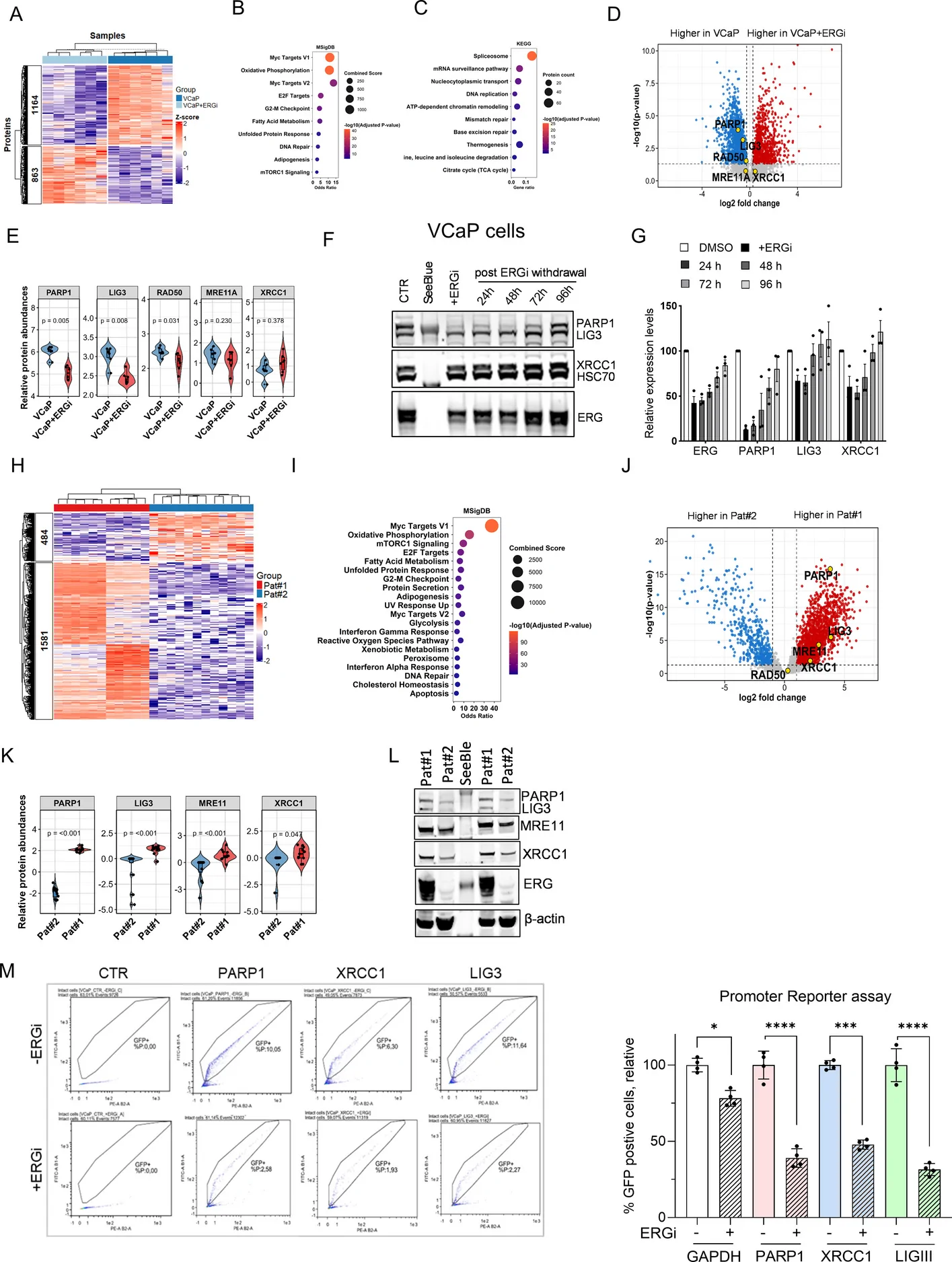
ERG inhibition induces distinct proteomic alterations in ERG-positive tumors. **A** Heatmap of differentially abundant proteins (two-sample t test, FDR, *p* < 0.05) in VCaP cells treated with vehicle control or ERGi. Red indicates higher relative abundance and blue indicates lower relative abundance. **B** Bubble plot of the top enriched MSigDB Hallmark pathways among the 1,164 proteins significantly reduced after ERG inhibition in VCaP cells. The x-axis shows the odds ratio, where values > 1 indicate overrepresentation in the pathway. The y-axis shows enriched terms. Circle color reflects -log_10_ adjusted p-value, and circle size represents the combined score. **C** Bubble plot of the top KEGG pathways enriched among the 1,164 proteins reduced after ERG inhibition in VCaP cells. The x-axis shows the gene ratio, and the y-axis shows enriched terms. Circle color indicates -log10 ​ adjusted p-value, with red indicating higher significance. **D** Volcano plot of protein abundance changes in VCaP versus VCaP + ERGi. Significant proteins are shown above the horizontal threshold line (FDR, *p* < 0.05), and vertical lines indicate the fold-change cutoff (≥ 1.2-fold). PARP1, LIG3, XRCC1, RAD50, and MRE11A are highlighted. **E** Violin plots showing the abundance of PARP1, LIG3, RAD50, MRE11A, and XRCC1 in VCaP cells with or without ERGi. Boxes indicate the median and interquartile range, and points represent individual replicates. P-values were calculated using two-sided Wilcoxon rank-sum tests. **F** Western blot analysis of the indicated proteins in VCaP cells before and after ERG inhibition, including a recovery condition 96 h after ERGi withdrawal. **G** Quantification of band intensities from F. HSC70 was used as a loading control. **H** Heatmap of differentially abundant proteins (two-sample t test, FDR, *p* < 0.05) between ERG-positive patient 1 (Pat#1) and ERG-negative patient 2 (Pat#2). Red indicates higher relative abundance and blue indicates lower abundance. **I** Bubble plot of significantly enriched MSigDB Hallmark pathways among proteins with higher abundance in Pat#1. **J** Volcano plot showing proteins more abundant in Pat#1 versus Pat#2 (FDR < 0.05, fold-change cutoff = 2). PARP1, LIG3, MRE11, and XRCC1 are highlighted. **K** Violin plots of PARP1, LIG3, MRE11A, and XRCC1 abundances in Pat#1 versus Pat#2. Each panel shows the distribution across replicates, median and interquartile range, and individual measurements. P-values were calculated using two-sided Wilcoxon rank-sum tests. **L** Western blot analysis of the indicated proteins in Pat#1 and Pat#2 (n = 2). β-actin was used as loading controls. **M** Left panel: Representative FACS images showing GFP-positive cells following transfection of ERGi-treated (+ ERGi) or untreated (-ERGi) VCaP cells with GLuc-ON™ reporters containing the promoters of the indicated genes. Right panel: Quantification of relative percentage of GFP-positive cells as an indicator of ERG-dependent promoter activity measured by GLuc-ON™ reporter assay in VCaP cells. Cells transfected with GAPDH, PARP1, XRCC1, or LIG3 promoter reporters were analyzed by flow cytometry 48 h later. Shown are means ± SD of four biological replicates. Statistical significance was determined using unpaired Mann–Whitney test (*P < 0.05, ***P < 0.001, ****P < 0.0001)

To elucidate the molecular mechanisms underlying ERG inhibition-mediated selective radiosensitization in ERG-positive cells, bottom-up differential quantitative proteomics was performed using LC–MS/MS in ERG-positive VCaP cells and ERG-negative DU145 and BPH-1 cells, both before and after ERG inhibition. In total, 5066 proteins were identified and quantified across all cell lines (Table S1). Distinguishable proteome profiles were identified across the studied cell lines before and after ERG inhibition, as visualized in the heatmap of 4343 significantly differentially abundant proteins (multiple sample tests ANOVA, FDR, *p* < 0.05; Fig. S6 A and Table S2). Pathway enrichment analysis (PEA) using KEGG and the MSigDB Hallmark gene set library, based on proteins with significantly reduced abundances after ERG inhibition (n = 1164 in VCaP, n = 1549 in DU145 and n = 1478 in BPH1 cells), identified DNA repair (MSigDB) and DNA replication (KEGG) among the top ten depleted pathways in VCaP cells (Fig. 4A-C. S6, A-G and Table S3). Among the downregulated DNA repair proteins identified in ERG-treated VCaP cells were PARP1-EJ-related proteins, including PARP1 (*p* = 0.005) and LIG3 (*p* = 0.008). XRCC1 showed a modest decrease that did not reach significance (*p* = 0.378). MRE11A, a key DNA end-resection factor, also trended toward lower abundance without statistical significance (*p* = 0.230), whereas RAD50 was significantly reduced (*p* = 0.031) (Fig. 4D&E and S6H). Western blot validation confirmed reduced PARP1 and LIG3 protein levels in VCaP cells following ERGi treatment (Fig. 4F&G). In contrast, DNA repair-related pathways were not among the top ten downregulated pathways in ERG-negative DU145 or BPH1 cells (Fig. S6B-G and Table S3).

To evaluate the clinical relevance of these findings, we performed a differential quantitative proteomic analysis on snap-frozen PCa biopsies from an ERG-positive tumor (Pat#1: two biopsies with 6 and 5 replicates, respectively) and an ERG-negative tumor (Pat#2: two biopsies with 6 replicates each). Based on the 2065 proteins with significant differences between Pat#1 and Pat#2 (Table S4), distinct proteomic profiles were observed (two sample tests, FDR *p* < 0.05) (Fig. 4H). PEA revealed significant upregulation of DNA repair pathways in the ERG-positive tumor (Fig. 4I and S7B), interestingly consistent with the proteomic profile previously observed in VCaP cells. Specifically, PARP1-EJ-related proteins including PARP1, LIG3 and XRCC1 were significantly more abundant in Pat#1 compared with Pat#2 (Fig. 4J&K and S7C), showing an expression pattern similar to that of VCaP cells. Notably, although MRE11 was not enriched in the proteomic data from ERG-positive VCaP cells, it was significantly upregulated in the ERG-positive Pat#1 proteomic dataset (Fig. 4J&K). Immunoblotting validated the upregulation of these proteins in the ERG-positive tumor sample from Pat #1 compared to the ERG-negative sample from Pat#2 (Fig. 4L). As further validation of the proteomics and immunoblotting data, we examined the effect of ERG inhibition on the promoter activity of PARP1, XRCC1, and LIG3 in VCaP cells using Gluc-ON promoter reporter assays. To this end, VCaP cells were transfected with plasmids containing the PARP1, XRCC1, LIG3, or GAPDH promoter upstream of a GFP reporter gene and were treated with or without ERGi. Notably, ERGi markedly reduced the promoter activity of PARP1 (~ 60%), XRCC1 (~ 52%), and LIG3 (~ 67%), as evidenced by the substantial decrease in GFP expression following ERG inhibition (Fig. 4M). These findings demonstrate that the transcriptional activation of these DNA repair genes in VCaP cells is ERG-dependent. Together, these findings suggest that ERG inhibition sensitizes ERG-positive PCa cells to IR, primarily through suppression of the PARP1-EJ repair pathway, as evidenced by (i) the downregulation of its players such as PARP1, XRCC1, and LIG3 following ERG inhibition and (ii) the differential expression patterns of these proteins in ERG-positive vs ERG-negative tumors.

### ERG inhibition induces selective radiosensitization in ERG-positive patient-derived PCa organoids (PDOs)

**Fig. 5:**
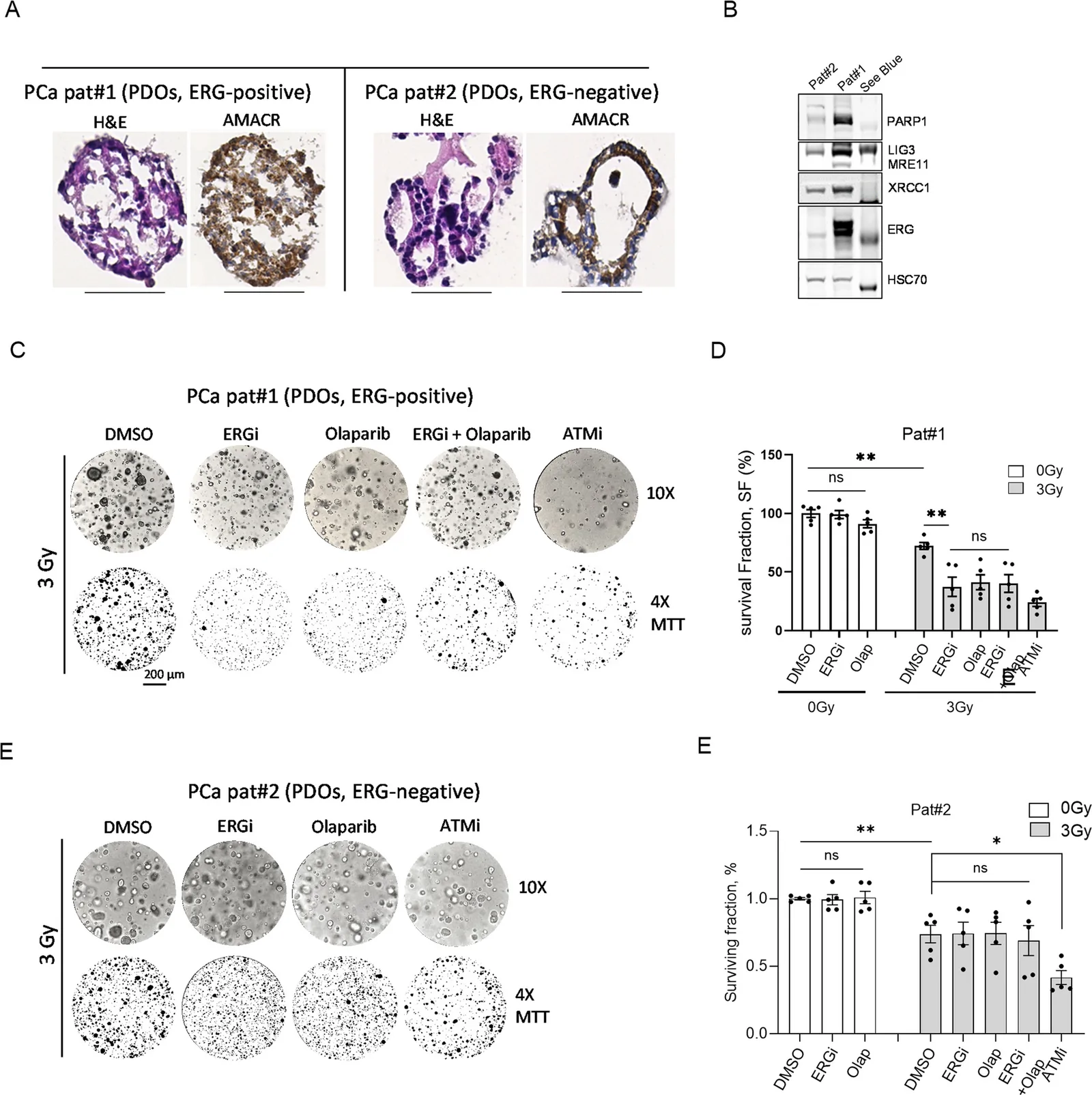
ERG inhibition radiosensitizes ERG-positive patient-derived PCa organoids. **A** Representative images of H&E staining (left) and immunohistochemical staining of the PCa marker AMACR (right) in PDOs established from the indicated PCa patients. Scale bar: 50 μm. **B** Western blot showing protein expression levels of ERG, and PARP1-EJ-related proteins (PARP1, LIG3, and XRCC1) in ERG-positive (Pat#1) and ERG-negative PCa PDOs (Pat#2). HSC70 was used as a loading control. **C** Upper panel: Representative images of 3D-colonies of PDOs from pat#1 treated with DMSO, ERGi, olaparib, or ATMi before 3 Gy irradiation; with 10X objective. Lower panel: 3D colonies of harvested and MTT-stained PDOs from Pat#1; with 4X objective. **D** Quantification of survival fractions from the data presented in C. **E** Upper panel: Representative images of 3D-colonies of ERG-negative PDOs from pat#2 following the indicated treatments. Lower panel: 3D colonies of harvested and MTT-stained PDOs from Pat#2. **F** Quantification of survival fractions from the data presented in E. All statistical significance was determined using unpaired Mann–Whitney test (*P < 0.05, **P < 0. 01); ns, not significant. The results are presented as the mean ± SEM of five independent experiments

Using ex vivo TSC, we demonstrated the selective inhibitory effect of ERGi on the repair capacity of IR-induced DSBs in ERG-positive cells, which is considered an indirect measure for radiosensitivity and radiosensitization. To directly assess the impact of ERG inhibition on radiosensitivity in clinically relevant models, we established PDOs from two PCa patients (one ERG-positive and one ERG-negative) as previously described [7]. Indeed, the generated PDOs recapitulated key features of PCa and were positive for the PCa-positive marker AMACR (Fig. 5A). Immunoblotting confirmed the ERG status of each PDO and notably revealed higher expression levels of the PARP1-EJ-related proteins in the ERG-positive PDO compared to the ERG-negative counterpart (Fig. 5B), consistent with our in vitro and ex vivo findings. To analyze the functional consequences of ERG inhibition on radiosensitivity, both PDO models were treated with 0.25 µM ERGi for 48 h prior to irradiation with 3 Gy and CFA was performed in a 3D setting to evaluate the survival fractions post treatment. Viable cells within harvested PDOs were stained with MTT to enhance visualization and accurate colony quantification (Fig. 5C-F). Compared to untreated controls, irradiation alone reduced survival by almost 30% in ERG-positive PDOs and 20% in ERG-negative PDOs. Treatment with ERGi alone did not affect the survival in either organoid model (Fig. S8 A&B). However, combining ERGi with 3 Gy resulted in a significant reduction in survival specifically in the ERG-positive PDO (63% reduction) (Fig. 5D), whereas the ERG-negative PDOs showed no further decrease compared to IR alone (Fig. 5F). Consistent with ERGi-mediated impairment of DSB repair in ERG-positive cells, ERG inhibition increased residual γH2AX and 53BP1 foci in PDOs from ERG-positive, but not ERG-negative, PCa patients (Fig. S8C&D). Furthermore, no additive or synergistic effect was observed following combining ERGi and the PARPi olaparib on the radiosensitivity (Fig. 5D&F), supporting the notion that both inhibitors converge on a common PARP1-EJ pathway to radiosensitize ERG-positive PCa cells. As a positive control, ATM inhibition robustly decreased cell survival in both ERG-positive and ERG-negative PDOs. Taken together, these preclinical findings demonstrate that ERG inhibition selectively enhances the radiosensitivity of ERG-positive PCa, highlighting a potential therapeutic strategy for molecularly stratified PCa treatment.

### Inhibition of ERG enhances the therapeutic efficacy of androgen receptor inhibitors in PCa

**Fig. 6:**
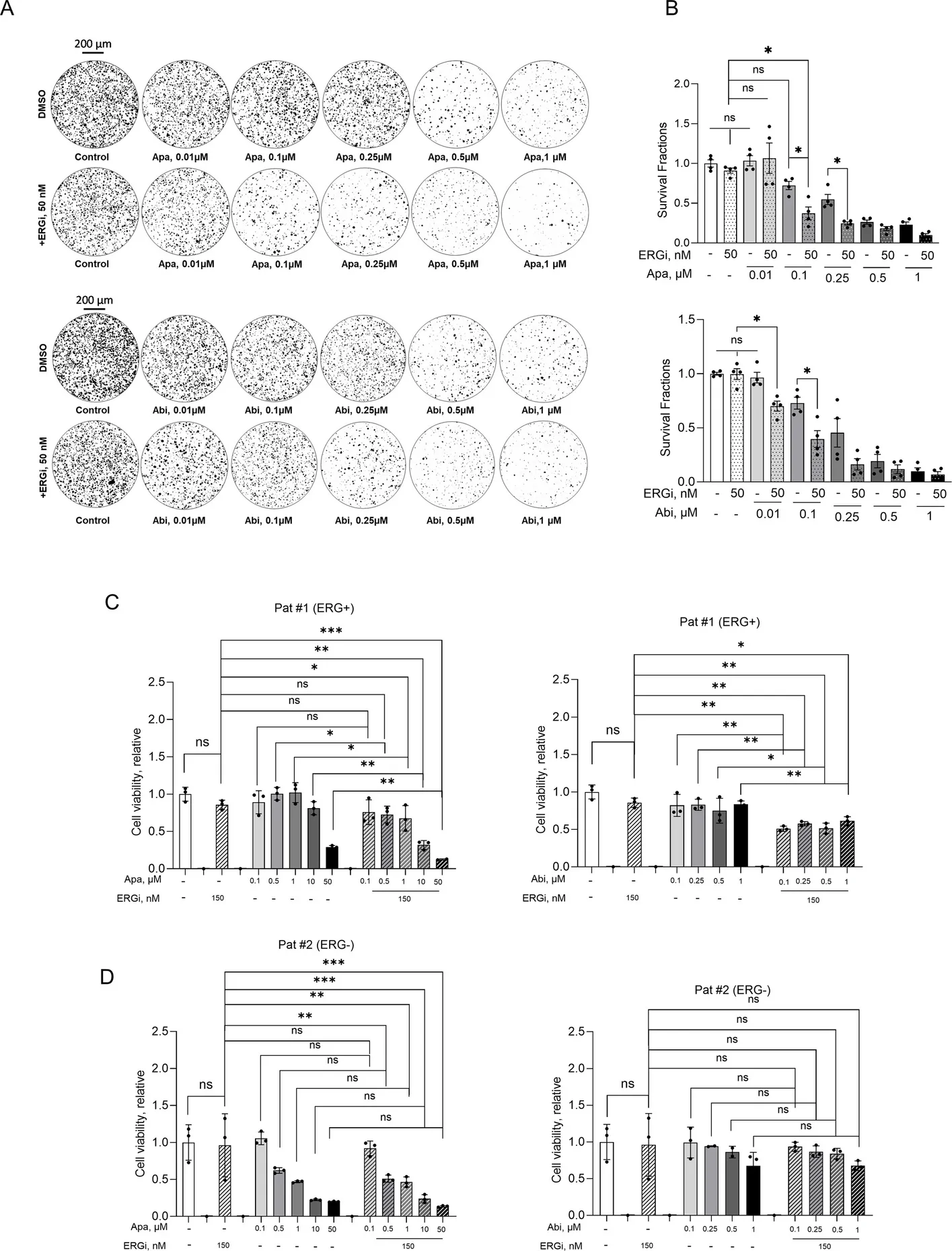
ERG inhibition enhances the efficacy of androgen receptor signaling inhibitors specifically in ERG-positive tumors. **A** Representative images of MTT-stained VCaP spheroids treated with the indicated concentrations of apalutamide (upper panel; Apa) or abiraterone (lower panel; Abi), either alone or in combination with 50 nM ERGi. **B** Clonogenic survival of VCaP cells treated with ERGi (50 nM) alone or in combination with apalutamide (upper panel; Apa) or abiraterone (lower panel; Abi) at the indicated concentrations. **C**&**D** CellTiter-Glo–based relative cell viability of PDOs derived from an ERG-positive patient (Pat #1) and an ERG-negative patient (Pat #2), treated with ERGi alone or in combination with Apa or Abi at the indicated concentrations. All data in this figure are presented as mean ± SEM from at least three independent experiments. All statistical significance was determined using unpaired Mann–Whitney test (*P < 0.05, **P < 0. 01, ***P < 0. 001); ns, not significant

**Fig. 7:**
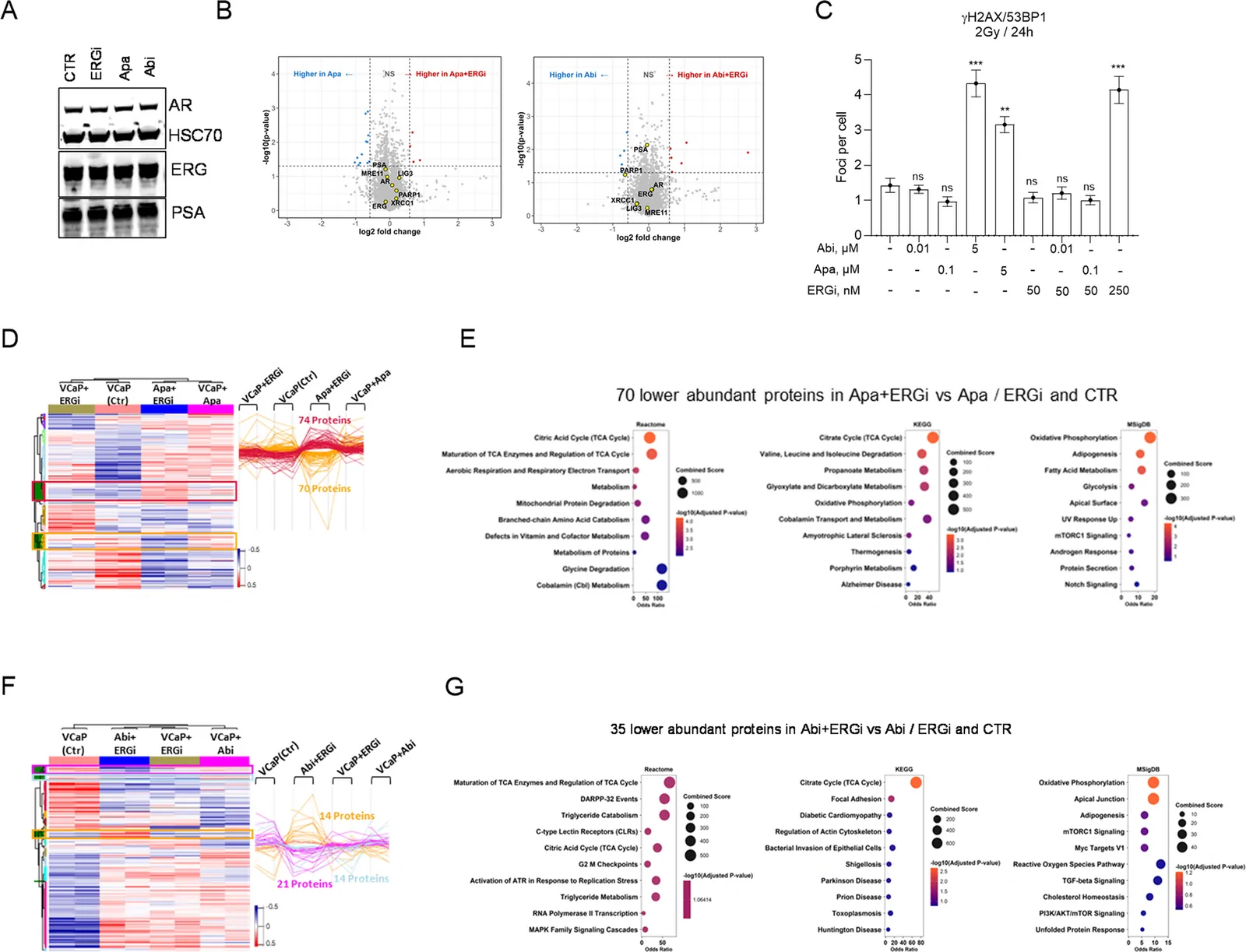
Low-dose combined ERG and AR pathway inhibition impairs prostate cancer cell survival without further suppression of canonical DNA repair and AR signaling. **A** Western blot analysis of the indicated proteins in VCaP cells after the indicated treatments. **B** Volcano plots showing differentially abundant proteins in VCaP cells treated with Apa versus Apa + ERGi and Abi versus Abi + ERGi. Proteins increased by combination treatment are shown in red, proteins decreased are shown in blue, and non-significant proteins are shown in grey (FDR *p* < 0.05, fold change cut-off = 1.5). PARP1, XRCC1, Ligase III, MRE11, ERG, AR and PSA are labelled. **C** Quantification of residual colocalized γH2AX/53BP1 foci per cell 24 h after 2 Gy irradiation in VCaP cells treated with the indicated concentrations of abiraterone (Abi), apalutamide (Apa), and ERGi. Shown are the means ± SD of foci measured in at least 50 cells. Statistical significance was determined using unpaired Mann–Whitney test (**P < 0. 01, ***P < 0. 001); ns, not significant. **D** Heatmap and clustering analysis of proteomic changes in VCaP cells treated with vehicle control, ERGi (50 nM), Apa (0.1 µM), or ERGi plus Apa (ANOVA test, FDR *p* < 0.05). The right panel shows the corresponding protein clusters and their trajectories across treatment conditions. **E** Bubble plots showing the top enriched Reactome, KEGG, and MSigDB hallmark pathways among the 70 proteins that are lower in Apa + ERGi than in Apa alone. **F** Heatmap and clustering analysis of proteomic changes in VCaP cells treated with vehicle control, Abi, or ERGi plus Abi. The right panel shows the corresponding protein clusters and their trajectories across treatment conditions. **G** Bubble plots showing top enriched Reactome, KEGG, and MSigDB hallmark pathways among the 35 proteins that are lower in Abi + ERGi than in Abi alone

ERG modulates the normal AR regulatory network in PCa by suppressing AR expression and inhibiting its transcriptional activity on canonical target genes. This appears to redirect AR-signaling from differentiation toward oncogenic, growth-promoting pathways, hence contributing to tumor progression [35, 36]. Notably, ERG and AR engage in a complex negative autoregulatory feedback loop that tightly controls their expression and functional balance, which is critical for the oncogenic program in ERG-positive PCa [37]. Therefore, simultaneous targeting of both AR and ERG could disrupt this oncogenic interplay more effectively than AR inhibition alone, offering a promising strategy to enhance therapeutic outcomes. To address this, we investigated the effect of combined ERGi and AR pathway inhibitors (ARPIs) on the survival of 3D VCaP spheroids. Specifically, spheroids derived from VCaP cells were treated with 50 nM ERGi for 48 h, followed by treatment with increasing concentrations of either apalutamide (Apa) or abiraterone (Abi) (0, 0.01, 0.1, 0.25, 0.5, and 1 µM). To limit potential ERGi-associated cytotoxicity, we reduced the ERGi concentration to one-fifth of the IC50 (i.e., 50 nM). This concentration did not exert a significant inhibitory effect on cell growth (Fig. S1B). As anticipated, 50 nM ERGi did not impair VCaP spheroid survival, whereas monotherapy with Apa or Abi elicited dose-dependent growth inhibition (Fig. 6A&B). Notably, combining ERGi with either ARPI markedly enhanced spheroid growth suppression relative to ARPI monotherapy (Fig. 6A&B). Survival analysis revealed that increasing concentrations of Apa or Abi, both alone and in combination with ERGi (50 nM), did not markedly reduce cell survival in ERG-negative/AR-negative DU145 or ERG-negative/AR positive LNCaP cells, with surviving fractions remaining above 50% in all treatment conditions and no clear synergistic growth inhibition observed with combined therapies (Fig. S9). Collectively, these findings support the hypothesis that dual targeting of ERG and AR-signaling could offer a promising treatment approach for PCa. We next validated these findings in PDOs from ERG-positive and ERG-negative PCa patients (Fig. 6C&D). In ERG-positive PDOs, ERGi at 150 nM (one fifth the IC50, data not shown) potentiated the cytotoxic effects of low-dose Apa and Abi, whereas ERG-negative PDOs showed no meaningful additional reduction in survival with the combination (Fig. 6C&D). Importantly, immunoblotting and proteomic profiling did not reveal further suppression of AR signaling or DNA damage repair proteins in the combination setting (Fig. 7A &B, Fig. S10A,and Table S6). Moreover, residual γH2AX/53BP1 foci after irradiation were not increased beyond the levels observed with single-agent treatment (Fig. 7C). Instead, the proteomic data suggest that the improved efficacy of combined ERGi and ARPI treatment reflects broader remodeling of tumor cell biology (Fig. 7D-G and Fig. S10B&C, Table S7). In ERG-positive models, dual treatment was associated with altered abundance of proteins linked to mitochondrial metabolism, oxidative phosphorylation, and the tricarboxylic acid cycle, together with pathways involved in respiratory electron transport and cellular stress adaptation. The enrichment patterns in the lower-abundant protein clusters are particularly consistent with reduced mitochondrial activity and diminished energetic flexibility, indicating that the combination may impose a metabolic constraint that compromises cell fitness rather than simply intensifying AR pathway blockade. Taken together, these findings support a context-specific therapeutic interaction between ERG and AR inhibition in ERG-positive prostate cancer and point to metabolic vulnerability as a plausible contributor to the observed response.

## Discussion

Our study highlights the multifaceted role of the transcription factor ERG in PCa, revealing its deep involvement in DNA repair pathway regulation and broader oncogenic programs. Rather than focusing on the debate about its role in PCa prognosis, our findings emphasize how ERG shapes DNA repair machinery, signaling networks, and therapeutic vulnerabilities that can be exploited for precision medicine. In vitro analysis using the ERG-overexpressing cell line VCaP, which carry the TMPRSS2:ERG fusion, demonstrated radiosensitization by ERG-inhibition, as shown by increased residual DSB foci, elevated levels of lethal chromosomal aberrations and micronucleated cells associated with mitotic catastrophe and decreased survival. The effects on DSB repair and survival were validated using ERGi-USU, which targets ribosomal ERG synthesis [30], VPC-18005, which blocks ERG-DNA binding [31], as well as ERG siRNA. These findings were further supported using PCa tissue slice cultures and PDOs, demonstrating that selective delay of DSBs damage signaling reduced survival only in ERG-overexpressing PCa samples or PDOs. Of note, this radiosensitization effect mediated by ERG targeting was selectively restricted to ERG-overexpressing tumors, while ERG-normal tumor cells and normal cells remained largely unaffected, underscoring a therapeutic window for precision targeting of ERG-driven PCa.

A key clinical challenge in PCa is the need to further improve RT outcomes, especially for patients with radioresistant disease or incomplete response to standard approaches. Our recent study highlights that a substantial subset of PCa tumors, characterized by elevated ERG and PARP1-EJ pathway gene expression, may be particularly suited for personalized combination strategies that enhance RT efficacy through PARP inhibition [7]. Here, our proteomic analyses revealed that ERG influences the abundance of multiple DNA repair-associated proteins. Notably, ERG-dependent changes were observed in key components of error-prone repair pathways, including PARP1, LIG3, and XRCC1 (associated with PARP1-EJ), MRE11, RAD50, and NBN (core MRN complex members involved in DNA-end resection and pathway switching). These findings align with previous studies highlighting the roles of these proteins in PCa DNA repair and tumor progression. PARP1, LIG3, and XRCC1 are essential components of the error-prone PARP1-EJ, contributing to genomic instability and potentially therapy resistance in PCa [38, 39]. Furthermore, the MRN complex proteins sense DNA DSB, initiate resection, and influence repair pathway choice, with elevated MRE11 levels associated with worse PCa outcomes [40]. Upregulation of PARP1-EJ pathway genes in ERG-positive tumors correlates with greater reliance on alternative end-joining for repair of IR-induced double-strand breaks. This molecular phenotype provides both a mechanistic rationale and a biomarker-driven pathway for patient stratification, suggesting that those with tumors showing high ERG and PARP1-EJ expression may derive the greatest benefit from ERG inhibitor plus RT or PARPi combinations. Importantly, robust patient selection is essential. TMPRSS2:ERG fusion and high ERG expression can be detected in patient biopsies or liquid assays [41], and combined with PARP1-EJ gene profiling allow for rational stratification. Not all ERG-positive tumors respond equally, owing to intertumoral heterogeneity and potential escape mechanisms, but the majority do benefit. This specificity not only enhances efficacy but also offers a tumor-selective strategy with limited toxicity to normal tissues. The presence of PARP1-EJ pathway activation in about a third of PCa cases [7], overlapping largely with ERG-positive tumors, provides a molecular basis for combining ERGi and IR.

The precise mechanism by which ERG regulates the expression of these genes remains to be fully elucidated. In the present study, we show that ERG influences the promoter activity of PARP1, XRCC1, and LIG3. However, it remains unclear whether this effect reflects a direct transcriptional action of ERG at these promoters or an indirect regulatory mechanism mediated through downstream effectors. This distinction is important, as ERG has previously been reported to control DNA repair gene expression through both direct and indirect mechanisms. Direct regulation involves recruitment of key DNA repair proteins such as PARP1 and DNA-PKcs to chromatin sites, thereby orchestrating transcriptional programs that favour alternative PARP1-EJ [42]. Indirectly, ERG drives broader oncogenic transcriptional networks, potentially influencing PARP1-EJ components via other transcription factors and cofactors. Additionally, ERG reorganizes chromatin architecture by engaging in long-range chromatin interactions that juxtapose enhancers with target gene promoters [43, 44], thereby facilitating coordinated activation of target gene programs, including DNA repair pathways such as PARP1-associated end-joining. Chromatin structure regulation thus emerges as an additional mechanism by which ERG promotes repair pathway switching toward PARP1-EJ. In our study, KEGG pathway enrichment analysis of proteins significantly downregulated after ERG inhibition in VCaP cells identified ATP-dependent chromatin remodeling as a top pathway. This suggests coordinated suppression of chromatin accessibility critical for DNA repair protein recruitment and pathway choice at DSBs [45]. Notably, emerging evidence indicates that ERG interacts with the histone methyltransferase EZH2 to modulate the epigenetic landscape and DNA repair gene expression [46, 47]. EZH2, the catalytic subunit of the Polycomb Repressive Complex 2 (PRC2), is frequently overexpressed in ERG-positive prostate tumors, where it drives chromatin remodeling, gene silencing, and therapy resistance [36]. Our proteomic data revealed EZH2 upregulation in ERG-positive cells, which was markedly reduced upon ERG inhibition (Fig. S6I). This observation aligns with previous findings showing that ERG directly binds and transcriptionally activates the EZH2 promoter [48]. Intriguingly, EZH2 reciprocally enhances ERG function through post-translational methylation at lysine 362 within its auto-inhibitory domain, thereby boosting ERG’s DNA-binding and transcriptional activity to form a positive feedback loop [48]. This ERG-EZH2 axis likely mediates ERG’s regulation of DNA repair by remodeling chromatin architecture to enhance accessibility and expression of DNA repair genes, including those involved in the PARP1-EJ.

Collectively, ERG likely regulates PARP1-EJ-related genes through a combination of direct DNA binding, control of epigenetic modifiers and transcriptional networks, modulation of DNA damage signaling, and alteration of chromatin architecture, promoting robust expression of alternative end-joining repair components critical for tumor survival.

Translational challenges remain, including refining RT techniques and fractionation, optimizing drug dosing and scheduling, and accounting for tumor heterogeneity, particularly in ERG expression and response to combination therapies. Short-course PARPi or ERGi during RT windows may mitigate resistance and limit toxicity, and biomarker-based stratification will help tailor treatment to those most likely to benefit.

Experimental ERG inhibitors such as VPC-18005 [31] and ERGi-USU [30] disrupt ERG function through distinct mechanisms, either by blocking ribosomal synthesis or interfering with DNA binding. Previous studies have shown that continuous treatment with ERGi-USU strongly suppresses tumor growth both in vitro and in mouse models [30]. However, prolonged ERGi exposure carries risks, including toxicity-related side effects and the development of drug resistance, which necessitates treatment discontinuation in clinical settings. We demonstrated here that ERG expression is fully restored within 120 h after drug withdrawal, suggesting that tumor cell growth is likely to recover as well, thereby raising concerns about therapeutic relapse following treatment interruption. To overcome the drawbacks of continuous ERG inhibition, we propose combining ERGi with radiotherapy. This strategy restricts ERGi exposure to the IR treatment window, minimizing toxicity and resistance while enhancing tumor cell kill. The current study provides the first evidence that ERGi markedly enhances IR efficacy specifically in ERG-positive PCa models, including in vitro cell lines, ex vivo TSCs, and PDOs.

Several critical aspects must be addressed to translate ERG-targeted therapy clinically. First, the development of tolerable ERG-specific inhibitors for phase 1 PCa trials remains a key barrier. TK216 is the sole ETS inhibitor that has reached early-phase clinical evaluation only in Ewing sarcoma (Clinical trial: NCT02657005) [49]. This reflects broader challenges in targeting transcription factors due to poor druggability (absent binding pockets, disordered domains, normal tissue toxicity risks) [49]. Second, patient selection requires validated ERG/ETS-fusion biomarkers to identify tumors that likely respond to ERG inhibition. Third, optimized aligned treatment scheduling with RT fractionation is critical. Based on the current finding that ERG expression recovers within 120 h post drug withdrawal, we propose ERGi administration 48 h before the first IR fraction, with subsequent dosing on day five (pre-weekend) to ensure suppression throughout the treatment course. This schedule minimizes systemic exposure while ensuring adequate ERG inhibition during critical DNA damage periods (i.e. by RT). The transient nature of our proposed treatment schedule (limited to the RT window) may substantially reduce toxicity concerns, yet dose-escalation studies of optimized derivatives remain essential. As a workaround, EZH2 inhibition emerges as a promising surrogate strategy, pending confirmation of EZH2's role in ERG-mediated DNA repair regulation (currently under investigation in our lab). EZH2 inhibitors (e.g., mevrometostat, tazemetostat and CPI-1205) are already in clinical trials for PCa and other solid tumors, with preclinical evidence showing disruption of ERG transcriptional networks, downregulation of DNA repair genes, and sensitization to genotoxic agents like RT. A transient schedule confined to the RT window could further mitigate toxicity, though dose-escalation studies of optimized agents remain essential.

ERG and AR extensively co-bind thousands of genomic sites, particularly enhancers, coordinating gene expression programs that drive tumor growth [36, 37]. This cooperative interaction creates a unique oncogenic transcriptional network in ERG-positive tumors, promoting proliferation and progression. Clinical studies also indicate that patients with tumors showing high combined ERG and AR expression have poorer prognosis [36], underscoring the biological and clinical relevance of their interaction and supporting the strategy of dual targeting.

In this context, our data provide direct preclinical support for combining ERGi with AR pathway inhibitors as a multipronged strategy in ERG-positive PCa. We showed here that in ERG positive tumors, ERGi at a non-cytotoxic concentration was ineffective as a single agent but significantly enhanced the growth-inhibitory effects of both apalutamide and abiraterone, whereas no comparable synergy was observed in ERG-negative cells. In line with this concept, previous studies showed that combining ARPIs (such as bicalutamide, abiraterone, or enzalutamide) with NOTCH inhibitors, which reduce both ERG and AR levels, enhances tumor growth inhibition and apoptosis while sparing ERG-negative cells [36, 50]. Importantly, the superior effect of combined ARPI and ERGi treatment was not explained by stronger suppression of canonical AR signaling or DNA repair. Instead, it was associated with reduced abundance of proteins involved in oxidative phosphorylation, the TCA cycle, and respiratory electron transport, suggesting the induction of an energetic deficit that may contribute to reduced cell viability. This interpretation is consistent with prior studies linking metabolic reprogramming to therapeutic vulnerability [51–53]. Together, these findings indicate that the interaction between ERG inhibition and AR pathway suppression is context dependent and largely restricted to ERG-positive models, supporting the idea that the therapeutic benefit arises from disruption of the ERG-AR oncogenic circuit.

## Conclusion

Collectively, our preclinical findings identify ERG as a master regulator of DNA repair pathways, supporting its clinical translation. ERG modulates the DNA repair machinery and interacts with AR signaling and EZH2, influencing tumor progression and therapeutic vulnerabilities. Targeting ERG, particularly in combination with irradiation or ARPIs, may disrupt tumor survival networks, slow disease progression, and reduce resistance development. Since nearly half of all PCa overexpress ERG, ERG-targeted therapies have broad clinical potential. Notably, while this study demonstrates ERGi-mediated enhancement of radiosensitivity and ARPIs sensitivity in ERG-positive PCa, it does not fully explore the long-term treatment effects, potential resistance mechanisms, or acquired adaptations that could limit clinical durability. Further research is needed to address these uncertainties, particularly in larger, more diverse patient cohorts, to validate the efficacy and safety of the proposed therapeutic strategies in clinical settings. The evolution of ERG from a molecular marker to a clinically actionable target marks a paradigm shift in PCa therapy, enabling precision medicine approaches based on DNA repair vulnerabilities.

## Supplementary Information

Supplementary Material 1.

Supplementary Material 2.

Supplementary Material 3.

Supplementary Material 4.

Supplementary Material 5.

Supplementary Material 6.

Supplementary Material 7.

Supplementary Material 8: Supplementary Fig. S1. Effect of ERGi on ERG expression and cell proliferation in prostate cancer cells. (A) Western Blot analysis showing ERG protein levels in panel of PCa cell lines VCaP, DU145 and PC3 and in the benign prostatic hyperplasia cell line BPH1. HSC70 was used as a loading control. (B) Proliferation assay demonstrating the inhibitory effects of increasing concentrations of ERGi on cell viability in the indicated cell lines. IC_50_ values are indicated. Data presented as mean±SEM of at least 3 independent experiments. (C) Upper panel: Representative western blot showing ERG protein levels in VCaP cells treated with different concentrations of ERGi ranging from 0.1 to 0.8 µM for 48 h. Lower panel: Quantification of the ERG expression from the upper panel. The intensities were measured and quantified using Licor Odyssey CLx imaging system (D) Upper panel: Schematic representation of the experiment, in which VCaP cells were treated with 0.25 µM for 48 h and subsequently released into ERGi-free medium. Middle panel: Western blot analysis of ERG protein levels following the indicated treatment scheme. Lower panel: Bar graph showing the quantification of the experiment in middle panel. Supplementary Fig. S2. (A) Quantification of gH2AX/53BP1 foci in VCaP, DU145 and BPH1 cells under the indicated treatment conditions. The number of foci after DMSO treatment was used to calculate the relative foci numbers in the indicated treatments. Shown are the means ± SEM of three independent experiments. Statistical significance was determined using unpaired Mann-Whitney test; ns, not significant. (B) Kinetic analysis of DSB foci resolution after irradiation. Quantification of γH2AX, 53BP1, and co-localized foci per cell in control and ERGi-treated VCaP cells following 2 Gy irradiation at the indicated time points. Data are shown as mean± SD of foci measured in at least 50 cells. Statistical significance was determined using unpaired Mann-Whitney test between individual treatments (*P< 0. 05, ****P < 0. 0001); ns, not significant. (C) Upper panel: Representative EdU-based cell cycle anaylsis of VCaP cells cultured in 2D setting at 12h, 24h and 48h after the indicated treatments. Middle Panel: Bar graphs showing the distribution of VCaP cells across cell cycle phases, including G1 (black), S (light grey), and G2 (white), following treatment with or without 250 nM ERGi for 48 h. Cells were subsequently exposed to 0 Gy or 2 Gy ionizing radiation and EdU was added 30 min prior to indicated time points pre fixation. Lower Panel: Table showing mean percentages for each cell cycle phase. (D) Same as in C, but using a 3D culture setting. EdU was added for 6 h prior to indicated time points. Data presented in C&D are shown as means ±SD of at least 3 biological replicates. (E) Upper panel: Representative metaphase preparation showing lethal chromosomal aberrations (arrows) in VCaP cells pretreated with 250 nM ERGi or mock treated (CTR) prior to irradiation with 2Gy. Metaphases were analyzed 24h after irradiation. Lower panel: Quantification of lethal chromosomal aberrations per metaphase for the experiment depicted in the upper panel. A total of 246 metaphases were analysed. (F) Upper panel: Representative immunofluorescence images of VCaP cells following the indicated treatments and stained with DAPI to visualize DNA. Micronuclei are indicated by arrows. Lower panel: Quantification of the percentage of micronucleated cells in the experiment shown in the upper panel. At least 500 cells were analyzed per treatment. Data presented in C-F are shown as means ±SD of at least 2 biological replicates. Statistical significance was determined using unpaired Mann-Whitney test comparing unirradiated cells to the indicated time point post-treatment (*P < 0. 05, (**P < 0. 01, (***P < 0. 001, ****P < 0. 0001); ns, not significant. Supplementary Fig. S3. ERG knockdown or blocking of the ERG-ETS domain inhibits the repair of IR-induced DSBs in VCaP cells. (A) Representative immunofluorescence images showing gH2AX (red), 53BP1 (green) in VCaP cells treated with 50 µM VPC-18005. Scale: 20µm. (B) Quantification of the data shown in A. Data shown are means ±SEM of 2 independent experiments. Statistical significance was determined using unpaired Mann-Whitney test (****P < 0. 0001); ns, not significant. (C) Western blot demonstrating ERG protein levels in VCaP cells after treatment with either 25 µM or 50 µM VPC-18005 for 48 h. (D) Western blot showing ERG protein levels in VCaP cells after the indicated time points post-transfection with ERG siRNA. (E) Western blot showing ERG expression in VCaP cells (i) untreated (DMSO), (ii) treated with ERG siRNA for 72 h, (iii) scrambled (iv) dual treatment with ERGi and ERG siRNA and (vi) treatment with 0.25 µM ERGi. (F) Representative immunofluorescence images for gH2AX (red) and 53BP1 (green) in VCaP cells under the indicated treatment conditions. Scale: 20µm. (G) Quantification of the experiment indicated in F. D stands for DMSO, sc for scrambeled RNA siE for siERG, and Ei for ERGi. Data are presented as means ±SEM of 2 independent experiments. Statistical significance was determined using unpaired Mann-Whitney test (**P < 0. 01); ns, not significant. Supplementary Fig. S4. (A) Representative immunofluorescence images showing gH2AX (red), 53BP1 (green) in DU145 (upper panel) and BPH1 (lower panel) cells following the indicated treatments. Scale: 20µm. (B&C) Quantification of the data shown in A. Shown are the means ±SEM of at least 3 independent experiments. Statistical significance was determined using unpaired Mann-Whitney test (****P < 0. 0001); ns, not significant. (D) Immunoblot analysis of PAR, PARP1, ERG and PSA in VCaP cells under the indicated treatments. Supplementary Fig. S5. (A & B) Representative 3D colonies generated from VCaP or DU145 cells following the indicated treatments. (C) Representative 2D colonies formed in BPH1 cells treated with ERGi (0.25 µM), ATMi (10 µM), either alone or combined prior irradiation with the indicated x-ray doses. (D) Survival fractions measured from the experiment presented in C. Data shown are means ± SEM from three independent experiments. (E) Cell viability of normal human fibroblasts (HF184) measured by CellTiter-Glo after irradiation in cells treated with DMSO or ERGi. (F) Quantification of gH2AX foci in TSCs from the indicated PCa patients at 24h post-3Gy before (DMSO) and after ERG inhibition (ERGi). Data shown are means ± SD of three biological replicates. Supplementary Fig. S6. Differential quantitative proteomics of ERGi-treated cells. (A) Heatmap visualization of significantly differentially abundant proteins (ANOVA-test, FDR, *p* < 0.05) across the cell lines before and after ERG inhibition. Z-scores (row-wise) are shown for each protein. Red indicates higher relative abundance, while blue represents lower relative abundance. Samples (in biological replicates, n=6) are grouped by cell line (VCaP, DU145, BPH1) and their ERG-inhibited counterparts (VCaP+ERGi, DU145+ERGi, BPH1+ERGi). (B&C) Heatmaps showing two-sample tests (FDR, *p* < 0.05) of changes in protein abundances in (B) DU145 ±ERGi and (C) BPH1±ERGi. (D-G) Bubble plots showing the top ten significantly enriched pathways from the MSigDB Hallmark gene set library in treated (C) DU145 (protein cluster size 1549) and (E) BPH1 (protein cluster size 1478), and from KEGG-enriched pathways in (F) DU145 and (G) BPH1. (H) Heatmap showing the expression of the indicated proteins in the studied cell lines before and after ERG inhibition. (I) Volcano plot showing log2 fold change in protein abundance (x-axis) against -log_10_ p-value (y-axis) of VCaP against VCaP+ERGi. Points above the horizontal dashed line represent proteins with significant changes in abundance (*p* < 0.05), while points outside the vertical dashed lines represent proteins with a fold change of at least 1.5 in abundance. EZH2 protein is depicted in gold. Supplementary Fig. S7. (A) Schematic illustration of the NIRL ablation setup. Snap-frozen patient tissue sections were placed on a cooling stage at -10°C inside a closed ablation chamber. The generated tissue aerosol was condensed and collected on a glass slide positioned opposite the ablation site. The collected material was solubilized in 1% SDC/TEAB buffer and processed for LC-MS/MS-based proteomic analysis. The ablated area measured 1 mm^2^, and each replicate yielded approximately 250 nL of collected material. (B) Bubble plot showing the top significantly enriched KEGG pathways identified from proteins with higher abundance in the ERG-positive patient sample (Pat #1) relative to the ERG-negative patient sample (Pat #2) (n=1,581 proteins). (C) Heatmap showing the relative abundance of the indicated proteins in the analyzed fresh-frozen patient tissue samples. Supplementary Fig. S8. (A&B) Representative images of PDOs derived from (A) ERG-positive and (B) ERG-negative PCa patients following the indicated treatment (upper panels; with 10X objective). Lower panels show the harvested MTT-stained PDOs: with 4X objective. (C&D) Quantification of gH2AX (red) and 53BP1 (green) foci in PDOs from (C) an ERG-positive (Pat. #1) and (D) an ERG-negative (Pat. #2) PCa patient, analyzed 1h (left panels) and 24 h (right panels) after 3 Gy irradiation following a 48-h pretreatment with DMSO or ERGi. Data are presented as the mean ± SD of at least 50 cells for each treatment. Supplementary Fig. S9: (A&B, left panels) Representative images of MTT-stained DU145 spheroids treated with the indicated concentrations of (A) abiraterone (Abi) or (B) apalutamide (Apa), either alone or in combination with 50nM ERGi. Right panels: Quantification of survival fractions determined by CFA based on the corresponding treatments shown in the left panels. (C&D, left panels) Representative images of MTT-stained LNCaP spheroids treated with the indicated concentrations of (A) Abi or (B) Apa, either alone or in combination with 50nM ERGi. Right panels: Quantification of survival fractions determined by CFA based on the corresponding treatments shown in the left panels. Data are presented as means ± SEM from two independent experiments. Supplementary Fig. S10. (A) Quantification of western blot analysis of AR, ERG, and PSA expression in VCaP cells treated with ERGi, Apa, or Abi. (B) Bubble plot showing the top enriched Reactome, KEGG, and MSigDB hallmark pathways among the 74 proteins that are higher in the Apa + ERGi condition compared with Apa alone. (C) Bubble plot showing the top enriched Reactome and KEGG hallmark pathways among the 14 proteins that are higher in the Abi+ERGi treatment condition compared with Abi alone.

## Data Availability

The mass spectrometry proteomics data have been deposited to the ProteomeXchange Consortium via the PRIDE [54] partner repository with the dataset identifiers PXD074487 and PXD082273. Reviewer access details (Token: IkfROrVuK0PL and ybv675iszGET). In addition to the proteomic data generated in this project from PCa patient tissue samples (Pat#1 and Pat#2, Table S4), the dataset comprised raw data from the VCaP, DU145, and BPH1 cell lines used in our previous work [7], as well as newly generated data from VCaP + ERGi, DU145 + ERGi, and BPH1 + ERGi in the present study. However, the data were processed using two separate Proteome Discoverer searches: one including only VCaP, DU145, and BPH1 for the previous study [7], and a second search including these cell lines together with their corresponding ERGi-treated conditions generated in this work (Table S1). Raw data files were reannotated as shown in Table S5.

## Abbreviations

2D/3D: Two-dimensional / Three-dimensional
53BP1: P53-binding protein 1
Abi: Abiraterone
ACN: Acetonitrile
ADT: Androgen deprivation therapy
AGC: Automatic gain control
AMACR: Alpha-methylacyl-CoA racemase
ANOVA: Analysis of variance
Apa: Apalutamide
AR: Androgen receptor
ARPIs: Androgen receptor signaling pathway inhibitors
ATM/ATMi: Ataxia telangiectasia mutated / ATM inhibitor
ATP: Adenosine triphosphate
BME: Basement membrane extract
CFA: Colony formation assay
DAPI: 4′,6-Diamidino-2-phenylindole
DDA: Data-dependent acquisition
DDR: DNA damage response
DMEM: Dulbecco's Modified Eagle Medium
DMSO: Dimethyl sulfoxide
DNA-PKcs/DNAPKi: DNA-dependent protein kinase catalytic subunit / DNA-PK inhibitor
DSBs: Double-strand breaks
DTT: Dithiothreitol
EdU: 5-Ethynyl-2′-deoxyuridine
eGFP/GFP: Enhanced green fluorescent protein / Green fluorescent protein
EJ: End joining
ERG/ERGi: ETS-related gene/ERG inhibitor
ESI: Electrospray ionization
ETS: E-twenty-six
EZH2: Enhancer of zeste homolog 2
FACS: Fluorescence-activated cell sorting
FA: Formic acid
FBS: Fetal bovine serum
FDR: False discovery rate
GAPDH: Glyceraldehyde 3-phosphate dehydrogenase
GLuc: *Gaussia* Luciferase
Gy: Gray (radiation unit)
H&E: Hematoxylin and eosin
HR/HRR: Homologous recombination / Homologous recombination repair
HSC70: Heat shock cognate 71 kDa protein
IAA: Iodoacetamide
IC10/IC50: 10% / 50% Inhibitory concentration
IF: Immunofluorescence
IR: Ionizing radiation
KEGG: Kyoto Encyclopedia of Genes and Genomes
LC–MS/MS: Liquid chromatography-tandem mass spectrometry
LIG3: DNA ligase 3
MRE11: Meiotic recombination 11
MRN: MRE11-RAD50-NBN complex
MSigDB: Molecular Signatures Database
MTT: 3-(4,5-Dimethylthiazol-2-yl)-2,5-diphenyltetrazolium bromide
NBN: Nibrin
NHEJ: Non-homologous end joining
NIRL: Nanosecond infrared laser
OCT: Optimal cutting temperature compound
PAR: Poly(ADP-ribose)
PARP1/PARPi: Poly(ADP-ribose) polymerase 1 / PARP inhibitor
PARP1-EJ: PARP1-dependent end joining
PBS: Phosphate-buffered saline
PCa: Prostate cancer
PDOs: Patient-derived organoid(s)
PEA: Pathway enrichment analysis
PFA: Paraformaldehyde
pEJ: Plasmid end joining
PRC2: Polycomb repressive complex 2
PRIDE: Proteomics Identifications Database
PSA/KLK3: Prostate-specific antigen / Kallikrein-3
RAD50: RAD50 double strand break repair protein
RPMI: Roswell Park Memorial Institute medium
RT: Radiotherapy
SD/SEM: Standard deviation / Standard error of the mean
SDC: Sodium deoxycholate
siRNA/scRNA: Small interfering RNA / Scrambled RNA
TCA: Tricarboxylic acid
TEAB: Triethylammonium bicarbonate
TMPRSS2: Transmembrane protease serine 2
TSCs: Tissue slice cultures
UPLC: Ultra-performance liquid chromatography
XRCC1: X-ray repair cross-complementing protein 1
γH2AX: Phosphorylated histone H2AX (Ser139)

## References

[1] Sung H, Ferlay J, Siegel RL, Laversanne M, Soerjomataram I, Jemal A, et al. Global cancer statistics 2020: GLOBOCAN estimates of incidence and mortality worldwide for 36 cancers in 185 countries. CA Cancer J Clin. 2021;71:209–49. 10.3322/caac.21660 33538338

[2] Evans AJ. Treatment effects in prostate cancer. Mod Pathol. 2018;31:S110-121. 10.1038/modpathol.2017.158 29297495

[3] Touma NJ, Izawa JI, Chin JL. Current status of local salvage therapies following radiation failure for prostate cancer. J Urol. 2005;173:373–9. 10.1097/01.ju.0000150627.68410.4d 15643174

[4] Ma TM, Chu FI, Sandler H, Feng FY, Efstathiou JA, Jones CU, et al. Local failure events in prostate cancer treated with radiotherapy: a pooled analysis of 18 randomized trials from the Meta-analysis of Randomized Trials in Cancer of the Prostate Consortium (LEVIATHAN). Eur Urol. 2022;82:487–98. 10.1016/j.eururo.2022.07.011 35934601

[5] Khanna KK, Jackson SP. DNA double-strand breaks: signaling, repair and the cancer connection. Nat Genet. 2001;27:247–54. 10.1038/85798 11242102

[6] Kötter A, Cornils K, Borgmann K, Dahm-Daphi J, Petersen C, Dikomey E, et al. Inhibition of PARP1-dependent end-joining contributes to Olaparib-mediated radiosensitization in tumor cells. Mol Oncol. 2014;8:1616–25. 10.1016/j.molonc.2014.06.008 PMC5528607 25028150

[7] Köcher S, Elsesy ME, Moustafa A, Mohammadi W, Perugachi Heinsohn A, Nagaraj Y, Oh-Hohenhorst SJ, Hahn J, Siebels B, Mair T, et al. Overexpression of the ERG oncogene in prostate cancer identifies candidates for PARP inhibitor-based radiosensitization. J Clin Invest 2026;136(6):e194949. 10.1172/JCI194949. PMC12987654 41632544

[8] Tomlins SA, Rhodes DR, Perner S, Dhanasekaran SM, Mehra R, Sun XW, et al. Recurrent fusion of TMPRSS2 and ETS transcription factor genes in prostate cancer. Science. 2005;310:644–8. 10.1126/science.1117679 16254181

[9] Soller MJ, Isaksson M, Elfving P, Soller W, Lundgren R, Panagopoulos I. Confirmation of the high frequency of the TMPRSS2/ERG fusion gene in prostate cancer. Genes Chromosomes Cancer. 2006;45:717–9. 10.1002/gcc.20329 16575875

[10] Mehra R, Tomlins SA, Shen R, Nadeem O, Wang L, Wei JT, et al. Comprehensive assessment of TMPRSS2 and ETS family gene aberrations in clinically localized prostate cancer. Mod Pathol. 2007;20:538–44. 10.1038/modpathol.3800769 17334343

[11] Minner S, Enodien M, Sirma H, Luebke AM, Krohn A, Mayer PS, et al. ERG status is unrelated to PSA recurrence in radically operated prostate cancer in the absence of antihormonal therapy. Clin Cancer Res. 2011;17:5878–88. 10.1158/1078-0432.CCR-11-1251 21791629

[12] Wang J, Cai Y, Ren C, Ittmann M. Expression of variant TMPRSS2/ERG fusion messenger RNAs is associated with aggressive prostate cancer. Cancer Res. 2006;66:8347–51. 10.1158/0008-5472.CAN-06-1966 16951141

[13] Nam RK, Sugar L, Yang W, Srivastava S, Klotz LH, Yang LY, et al. Expression of the TMPRSS2:ERG fusion gene predicts cancer recurrence after surgery for localised prostate cancer. Br J Cancer. 2007;97:1690–5. 10.1038/sj.bjc.6604054 PMC2360284 17971772

[14] Demichelis F, Fall K, Perner S, Andrén O, Schmidt F, Setlur SR, et al. TMPRSS2:ERG gene fusion associated with lethal prostate cancer in a watchful waiting cohort. Oncogene. 2007;26:4596–9. 10.1038/sj.onc.1210237 17237811

[15] Hoogland AM, Jenster G, van Weerden WM, Trapman J, van der Kwast T, Roobol MJ, et al. ERG immunohistochemistry is not predictive for PSA recurrence, local recurrence or overall survival after radical prostatectomy for prostate cancer. Mod Pathol. 2012;25:471–9. 10.1038/modpathol.2011.176 22080055

[16] Gopalan A, Leversha MA, Satagopan JM, Zhou Q, Al-Ahmadie HA, Fine SW, et al. TMPRSS2-ERG gene fusion is not associated with outcome in patients treated by prostatectomy. Cancer Res. 2009;69:1400–6. 10.1158/0008-5472.CAN-08-2467 PMC3676271 19190343

[17] Elsesy ME, Oh-Hohenhorst SJ, Löser A, Oing C, Mutiara S, Köcher S, Meien S, Zielinski A, Burdak-Rothkamm S, Tilki D, et al. Second-generation antiandrogen therapy radiosensitizes prostate cancer regardless of castration state through inhibition of dna double strand break repair. Cancers (Basel). 2020;12(9):2467. 10.3390/cancers12092467. PMC7563746 32878283

[18] Mansour WY, Borgmann K, Petersen C, Dikomey E, Dahm-Daphi J. The absence of Ku but not defects in classical non-homologous end-joining is required to trigger PARP1-dependent end-joining. DNA Repair (Amst). 2013;12:1134–42. 10.1016/j.dnarep.2013.10.005 24210699

[19] Rothkamm K, Christiansen S, Rieckmann T, Horn M, Frenzel T, Brinker A, et al. Radiosensitisation and enhanced tumour growth delay of colorectal cancer cells by sustained treatment with trifluridine/tipiracil and X-rays. Cancer Lett. 2020;493:179–88. 10.1016/j.canlet.2020.08.038 32891715

[20] Köcher S, Beyer B, Lange T, Nordquist L, Volquardsen J, Burdak-Rothkamm S, et al. A functional ex vivo assay to detect PARP1-EJ repair and radiosensitization by PARP-inhibitor in prostate cancer. Int J Cancer. 2019;144:1685–96. 10.1002/ijc.32018 30478958

[21] Elsesy ME, Oh-Hohenhorst SJ, Oing C, Eckhardt A, Burdak-Rothkamm S, Alawi M, et al. Preclinical patient-derived modeling of castration-resistant prostate cancer facilitates individualized assessment of homologous recombination repair deficient disease. Mol Oncol. 2023;17:1129–47. 10.1002/1878-0261.13382 PMC10257417 36694344

[22] Elsesy ME, Moustafa A, Oh-Hohenhorst S-J, Müller C, Alawi M, Mair T, Siebels B, Hu Z, Hahn J, Burdak-Rothkamm S, et al. Functional and omics-based rationale for the induction of BRCAness by androgen receptor pathway inhibitors to sensitize prostate cancer to PARP inhibition, regardless of HRR status. Br J Cancer. 2026;135(7):1140–51. 10.1038/s41416-026-03518-7. PMC13578762 42414479

[23] Hoeller U, Borgmann K, Bonacker M, Kuhlmey A, Bajrovic A, Jung H, et al. Individual radiosensitivity measured with lymphocytes may be used to predict the risk of fibrosis after radiotherapy for breast cancer. Radiother Oncol. 2003;69:137–44. 10.1016/j.radonc.2003.10.001 14643950

[24] Ramadan WS, Ahmed SBM, Talaat IM, Lozon L, Mouffak S, Gemoll T, et al. The histone acetyltransferase CBP participates in regulating the DNA damage response through ATM after double-strand breaks. Genome Biol. 2025;26:89. 10.1186/s13059-025-03528-3 PMC11980100 40200339

[25] Stadlhofer R, Moritz M, Fuh MM, Heeren J, Zech H, Clauditz TS, Schlüter H, Betz CS, Eggert D, Böttcher A, Hahn J. Lipidome analysis of oropharyngeal tumor tissues using nanosecond infrared laser (nirl) tissue sampling and subsequent mass spectrometry. Int J Mol Sci. 2023;24(9):7820. 10.3390/ijms24097820. PMC10178251 37175533

[26] Tyanova S, Temu T, Sinitcyn P, Carlson A, Hein MY, Geiger T, et al. The Perseus computational platform for comprehensive analysis of (prote)omics data. Nat Methods. 2016;13:731–40. 10.1038/nmeth.3901 27348712

[27] Wu T, Hu E, Xu S, Chen M, Guo P, Dai Z, et al. ClusterProfiler 4.0: a universal enrichment tool for interpreting omics data. Innovation (N Y). 2021;2:100141. 10.1016/j.xinn.2021.100141 PMC8454663 34557778

[28] Kanehisa M, Furumichi M, Sato Y, Matsuura Y, Ishiguro-Watanabe M. KEGG: biological systems database as a model of the real world. Nucleic Acids Res. 2025;53:D672-d677. 10.1093/nar/gkae909 PMC11701520 39417505

[29] Liberzon A, Birger C, Thorvaldsdóttir H, Ghandi M, Mesirov JP, Tamayo P. The Molecular Signatures Database (MSigDB) hallmark gene set collection. Cell Syst. 2015;1:417–25. 10.1016/j.cels.2015.12.004 PMC4707969 26771021

[30] Mohamed AA, Xavier CP, Sukumar G, Tan SH, Ravindranath L, Seraj N, et al. Identification of a small molecule that selectively inhibits ERG-positive cancer cell growth. Cancer Res. 2018;78:3659–71. 10.1158/0008-5472.CAN-17-2949 29712692

[31] Butler MS, Roshan-Moniri M, Hsing M, Lau D, Kim A, Yen P, et al. Discovery and characterization of small molecules targeting the DNA-binding ETS domain of ERG in prostate cancer. Oncotarget. 2017;8:42438–54. 10.18632/oncotarget.17124 PMC5522078 28465491

[32] Borowicz S, Van Scoyk M, Avasarala S, Karuppusamy Rathinam MK, Tauler J, Bikkavilli RK, Winn RA. The soft agar colony formation assay. J Vis Exp. 2014;(92):e51998. 10.3791/51998. PMC4353381 25408172

[33] Wang LH. Molecular signaling regulating anchorage-independent growth of cancer cells. Mt Sinai J Med. 2004;71:361–7. 15592654

[34] Mansour WY, Schumacher S, Rosskopf R, Rhein T, Schmidt-Petersen F, Gatzemeier F, et al. Hierarchy of nonhomologous end-joining, single-strand annealing and gene conversion at site-directed DNA double-strand breaks. Nucleic Acids Res. 2008;36:4088–98. 10.1093/nar/gkn347 PMC2475611 18539610

[35] Chen Y, Sawyers CL. Coordinate transcriptional regulation by ERG and androgen receptor in fusion-positive prostate cancers. Cancer Cell. 2010;17:415–6. 10.1016/j.ccr.2010.04.022 PMC2931314 20478521

[36] Yu J, Yu J, Mani RS, Cao Q, Brenner CJ, Cao X, et al. An integrated network of androgen receptor, polycomb, and TMPRSS2-ERG gene fusions in prostate cancer progression. Cancer Cell. 2010;17:443–54. 10.1016/j.ccr.2010.03.018 PMC2874722 20478527

[37] Wasmuth EV, Hoover EA, Antar A, Klinge S, Chen Y, Sawyers CL. Modulation of androgen receptor DNA binding activity through direct interaction with the ETS transcription factor ERG. Proc Natl Acad Sci U S A. 2020;117:8584–92. 10.1073/pnas.1922159117 PMC7165421 32220959

[38] Li X, Baek G, Ramanand SG, Sharp A, Gao Y, Yuan W, et al. BRD4 promotes DNA repair and mediates the formation of TMPRSS2-ERG gene rearrangements in prostate cancer. Cell Rep. 2018;22:796–808. 10.1016/j.celrep.2017.12.078 PMC5843368 29346775

[39] Alblihy A, Ali R, Algethami M, Shoqafi A, Toss MS, Brownlie J, et al. Targeting Mre11 overcomes platinum resistance and induces synthetic lethality in XRCC1 deficient epithelial ovarian cancers. NPJ Precis Oncol. 2022;6:51. 10.1038/s41698-022-00298-0 PMC9296550 35853939

[40] Wang J, Xu WH, Wei Y, Zhu Y, Qin XJ, Zhang HL, et al. Elevated MRE11 expression associated with progression and poor outcome in prostate cancer. J Cancer. 2019;10:4333–40. 10.7150/jca.31454 PMC6691708 31413753

[41] Alahdal M, Perera RA, Moschovas MC, Patel V, Perera RJ. Current advances of liquid biopsies in prostate cancer: molecular biomarkers. Mol Ther Oncolytics. 2023;30:27–38. 10.1016/j.omto.2023.07.004 PMC10415624 37575217

[42] Brenner JC, Ateeq B, Li Y, Yocum AK, Cao Q, Asangani IA, et al. Mechanistic rationale for inhibition of poly(ADP-ribose) polymerase in ETS gene fusion-positive prostate cancer. Cancer Cell. 2011;19:664–78. 10.1016/j.ccr.2011.04.010 PMC3113473 21575865

[43] Han S, Brenner JC, Sabolch A, Jackson W, Speers C, Wilder-Romans K, et al. Targeted radiosensitization of ETS fusion-positive prostate cancer through PARP1 inhibition. Neoplasia. 2013;15:1207–17. 10.1593/neo.131604 PMC3819636 24204199

[44] Zhang Z, Chng KR, Lingadahalli S, Chen Z, Liu MH, Do HH, et al. An AR-ERG transcriptional signature defined by long-range chromatin interactomes in prostate cancer cells. Genome Res. 2019;29:223–35. 10.1101/gr.230243.117 PMC6360806 30606742

[45] Price BD, D’Andrea AD. Chromatin remodeling at DNA double-strand breaks. Cell. 2013;152:1344–54. 10.1016/j.cell.2013.02.011 PMC3670600 23498941

[46] Yang YA, Yu J. EZH2, an epigenetic driver of prostate cancer. Protein Cell. 2013;4:331–41. 10.1007/s13238-013-2093-2 PMC4131440 23636686

[47] Liao Y, Chen CH, Xiao T, de la Peña Avalos B, Dray EV, Cai C, Gao S, Shah N, Zhang Z, Feit A, et al. Inhibition of EZH2 transactivation function sensitizes solid tumors to genotoxic stress. Proc Natl Acad Sci USA. 2022;119(3):e2105898119. 10.1073/pnas.2105898119. PMC8784159 35031563

[48] Zoma M, Curti L, Shinde D, Albino D, Mitra A, Sgrignani J, et al. EZH2-induced lysine K362 methylation enhances TMPRSS2-ERG oncogenic activity in prostate cancer. Nat Commun. 2021;12:4147. 10.1038/s41467-021-24380-6 PMC8260656 34230470

[49] Meyers PA, Federman N, Daw N, Anderson PM, Davis LE, Kim A, et al. Open-label, multicenter, phase I/II, first-in-human trial of TK216: a first-generation EWS::FLI1 fusion protein antagonist in Ewing sarcoma. J Clin Oncol. 2024;42:3725–34. 10.1200/JCO.24.00020 PMC11521759 38954782

[50] Chen Y, Chi P, Rockowitz S, Iaquinta PJ, Shamu T, Shukla S, et al. ETS factors reprogram the androgen receptor cistrome and prime prostate tumorigenesis in response to PTEN loss. Nat Med. 2013;19:1023–9. 10.1038/nm.3216 PMC3737318 23817021

[51] Nong S, Han X, Xiang Y, Qian Y, Wei Y, Zhang T, et al. Metabolic reprogramming in cancer: mechanisms and therapeutics. MedComm (Beijing). 2020;4:e218. 10.1002/mco2.218 PMC10041388 36994237

[52] McCann C, Kerr EM. Metabolic reprogramming: a friend or foe to cancer therapy? Cancers (Basel). 2021;13(13):3351. 10.3390/cancers13133351. PMC8267696 34283054

[53] Yoshida GJ. Metabolic reprogramming: the emerging concept and associated therapeutic strategies. J Exp Clin Cancer Res. 2015;34:111. 10.1186/s13046-015-0221-y PMC4595070 26445347

[54] Perez-Riverol Y, Bai J, Bandla C, García-Seisdedos D, Hewapathirana S, Kamatchinathan S, et al. The PRIDE database resources in 2022: a hub for mass spectrometry-based proteomics evidences. Nucleic Acids Res. 2022;50:D543-d552. 10.1093/nar/gkab1038 PMC8728295 34723319

